# An atom-economical addition of methyl azaarenes with aromatic aldehydes via benzylic C(sp^3^)–H bond functionalization under solvent- and catalyst-free conditions

**DOI:** 10.3762/bjoc.16.259

**Published:** 2020-12-23

**Authors:** Divya Rohini Yennamaneni, Vasu Amrutham, Krishna Sai Gajula, Rammurthy Banothu, Murali Boosa, Narender Nama

**Affiliations:** 1 Catalysis and Fine Chemicals Division, CSIR-Indian Institute of Chemical Technology, Hyderabad, Telangana, 500 007, India; 2 Academy of Scientific and Innovative Research, CSIR-HRDC Campus, Sector 19, Kamala Nehru Nagar, Ghaziabad, UP-201002, India

**Keywords:** aldehydes, azaarenes, benzylic addition, green chemistry, solvent-free conditions

## Abstract

A convenient practical approach for the synthesis of 2-(pyridin-2-yl)ethanols by direct benzylic addition of azaarenes and aldehydes under catalyst- and solvent-free conditions is reported. This reaction is metal-free, green, and was carried out in a facile operative environment without using any hazardous transition metal catalysts or any other coupling reagents. Different aromatic aldehydes and azaarenes were monitored, and the yields of the resulting products were moderate to excellent. We accomplished several azaarene derivatives under neat conditions through a highly atom-economical pathway. To evaluate the preparative potential of this process, gram-scale reactions were performed up to a 10 g scale.

## Introduction

Azaarenes are a distinct class of heterocyclic compounds possessing wide compatibility in the field of synthetic organic chemistry. The recent advancements in nitrogen-containing carbon compounds have marked them as an unusual moiety due to their attractive applications in biology and as materials [1–4]. Among various nitrogen-containing heterocyclic compounds, pyridine and quinolines are readily found in bioactive compounds [5]. The functionalization of alkylpyridines and quinolines is significant and plays a remarkable role in the efficient drug design [6–8]. Due to their conformational diversity, these compounds constitute a motif in various natural alkaloid products, such as chimanine and those derived from lobelia, sedum, etc. These compounds act as anti-HIV and anti-asthma drugs [9–11]. Mainly, 2-substituted quinolines and their analogues exhibits magnificent bioactivity [12–13]. The synthesis of higher cores of nitrogen-containing heterocyclic compounds through C(sp^3^)–H functionalization of simple compounds like methyl azaarenes allows direct transformation without any critical reaction conditions. Thus, in this regard the further development of this approach is still in need to be developed [14].

**Scheme 1:**
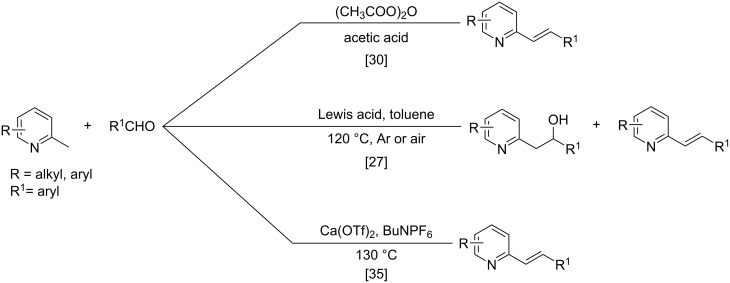
Benzylic addition of aldehydes to azaarenes using different catalysts.

**Scheme 2:**
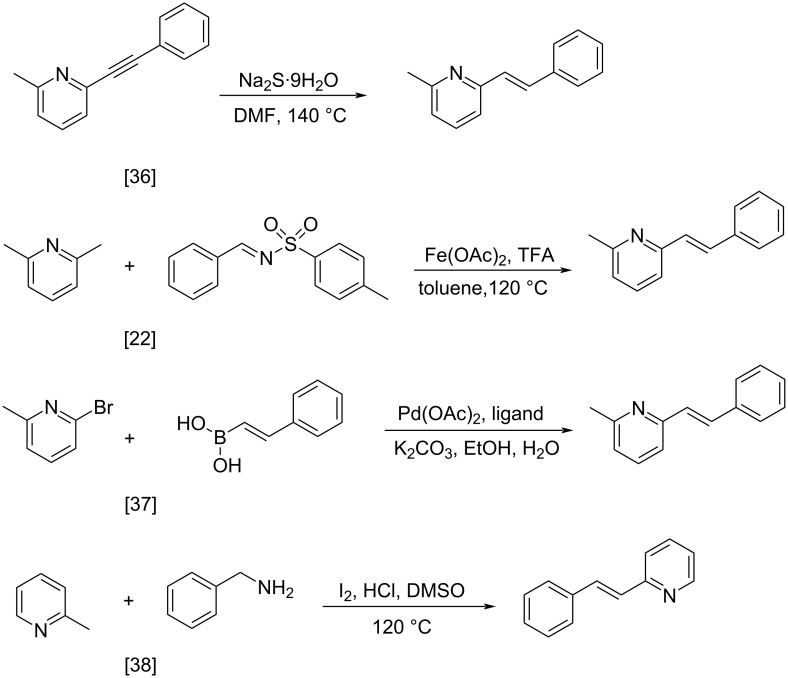
Synthesis of azaarene derivatives from different precursors.

The formation of new C–C bonds through direct C–H bond functionalization in organic chemistry is attractive [15]. Such methodologies are omnipresent and facilitate sustainable organic transformations for the synthesis of complex natural products and pharmaceuticals. In the past years, C−H bond functionalization catalyzed by transition metals received a strong emphasis, and other different catalytic systems have also been encouraged [16–20]. Huang and co-workers first realized the addition of alkylazaarenes directly to unsaturated bonds via C(sp^3^)–H functionalization [21–22]. The synthesis of pyridine and related azaarene derivatives involve the C(sp^3^)–H activation of 2-methylpyridines using different transition-metal compounds, Lewis acids, and Brønsted acids [23–28]. Recently, C(sp^3^)–H functionalizations of methylazaarenes with isatins and malononitrile under catalyst-free conditions have been reported [29]. Wang et al. reported the functionalization of benzylic C–H bonds of 2-methylazaarenes by nucleophilic addition to aromatic aldehydes catalyzed by acetic acid using harmful chlorinated solvent, and this reaction suffers from longer reaction times [30]. Rao et al. performed similar reactions without catalyst under microwave irradiation in the presence of water as a solvent [31], but when considering an industrial scale, there are numerous factors that serves as obstacles for the usage of microwave reactors, such as escalated heat loss, variations in the absorption, an inadequate penetrating ability of the radiation into the reaction medium, and further reflection of the microwaves. Nevertheless, the solvent was water, which required long extraction processes compared to solvent-free conditions. Castro and co-workers have also reported the synthesis of 1-phenyl-2-(2-pyridyl)ethanol and 1-phenyl-2-(2-pyridyl)ethene under catalyst- and solvent-free conditions. Despite the low yield of the product of around 4%, this work described crystallographic data and the influence of reaction conditions such as the temperature and the reaction time on the stability of 1-phenyl-2-(2-pyridyl)ethanol [32]. Different catalysts such as ionic liquids [33], CuFe_2_O_4_ [34], and Ca(OTf)_2_ [35] were also utilized. Due to the abundant importance of these products, several different approaches were reported (Scheme 1 and Scheme 2). These higher azaarene products were also achieved from other starting materials, such as 2-methyl-6-(phenylethynyl)pyridine [36], 2,6-dimethylpyridine (**1a**) and (*E*)-*N*-benzylidene-4-methylbenzenesulfonamide [22], 2-bromo-6-methylpyridine and (*E*)-styrylboronic acid [37], as well as 2-methylpyridine and phenylmethanamine [38]. Most recently, Yaragorla et al. published a review on C(sp^3^)–H bond functionalization of 2-methylazaarenes [39]. These strategies are proficient, but due to the involvement of drastic reaction conditions, the use of expensive reagents, toxic metals, harmful solvents, and tedious workup procedures, they need to be reevaluated. Therefore, alternatives with environmentally benign reagents are much in focus.

Correspondingly, considering the exemplar shift from conventional synthetic methodologies towards green chemistry, there have been some alternatives or replacements of toxic catalysts and hazardous solvents in chemical reactions [40]. These conversions play a vital role for the syntheses of active pharmaceutical ingredients as these approaches reduce the contamination in an industrial setting. In addition, the replacement of toxic reagents by environmentally benign reagents can decrease pollution to some extent [41–42].

**Scheme 3:**
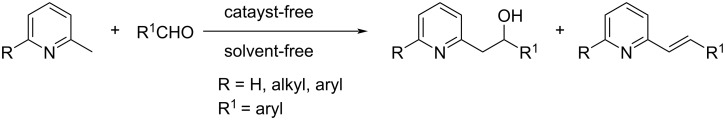
Our work: catalyst- and solvent-free benzylic addition of aldehydes to azaarenes.

However, to develop advantageous eco-friendly, atom-economical, simple, and efficient synthetic-organic processes under solvent- and catalyst-free conditions in order to synthesize highly demanding pharmaceuticals or natural products can be quite backbreaking [43–45]. From this perspective, we herein disclose environmentally benign, atom-economical, catalyst-free nucleophilic additions of benzylic-like azaarene C–H groups to various benzaldehydes under neat conditions (Scheme 3).

## Results and Discussion

With the purpose to develop an environmentally benign process, we first examined *p*-nitrobenzaldehyde (**2a**) and 2,6-dimethylpyridine (**1a**) as model substrates. Our investigation started with the reaction of *p*-nitrobenzaldehyde (**2a**) and 2,6-dimethylpyridine (**1a**) in the presence of Hβ zeolite as a catalyst at 120 °C in toluene for 24 h in a sealed vial (Table 1, entry 1). However, the desired compound **3a** was observed in traces. We thought that the catalyst pore size may be an obstruction to a higher yield and used a mesoporous material, MCM-41, as a catalyst, which slightly improved the yield (Table 1, entry 2). At the same time, increasing the temperature to 130 °C enhanced the yield of the product **3a** (Table 1, entry 3). In order to address the economic cost and ecological impact, we tried this reaction without using a solvent (Table 1, entry 4). Further, this reaction was screened using various zeolites, such as Hβ, H-mordenite, H-ZSM, HY, NaY, and MCM-41 under solvent-free conditions (Table 1, entries 5–9). In addition, to justify the role of the catalyst, this reaction was carried out in the absence of a catalyst. Thankfully, the yield of the product enhanced to 79% (Table 1, entry 10). We managed to increase the yield of the desired product **3a** by further screening of the reaction conditions under catalyst- as well as solvent-free conditions. As we increased the temperature to 135 °C, the yield of the product increased to 84% (Table 1, entry 11). An additional increase of the temperature to 140 °C did not exhibit any promising change, but decreasing the temperature to 120 °C reduced the yield of the product **3a** (Table 1, entries 12 and 13). Hence, we carried out further reactions at 135 °C. Generally, the temperature of the reaction plays a crucial role, in formation of a uniform reaction mixture of the reactants under solvent-free conditions. Increasing the reaction time did not produce a remarkable change, but when decreasing the reaction time, a drastic effect on the yield of the product **3a** was observed (Table 1, entries 14 and 15). Surprisingly, when the reaction was carried out in an open atmosphere, the yield of the required product **3a** was drastically reduced (Table 1, entry 16). Different reactant stoichiometries were analyzed to maximize the yield of the desired product **3a** (Table 1, entries 17–20). After these tests, we concluded the best yield of **3a** to be 92% (Table 1, entry 20), which was achieved from a reaction mixture of 2 mmol 2,6-dimethylpyridine (**1a**) and 1 mmol *p*-nitrobenzaldehyde (**2a**) at 135 °C for 24 h in a sealed vial without any catalysts or additive under solvent-free conditions.

**Table 1:** Optimization of the reaction conditions.^a^

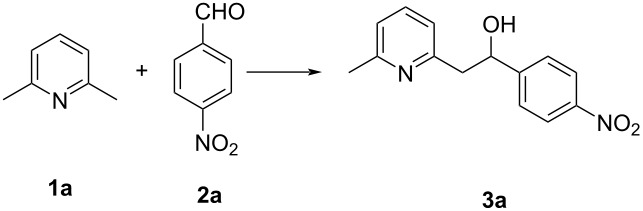				

entry	catalyst	solvent	temperature (°C)	yield (%)^b^

1	Hβ	toluene	120	4
2	MCM-41	toluene	120	10
3	MCM-41	toluene	130	23
4	MCM-41	—	130	62
5	Hβ	—	130	76
6	H-mordenite	—	130	38
7	H-ZSM	—	130	72
8	HY	—	130	30
9	NaY	—	130	6
10	—	—	130	79
11	—	—	135	84
12	—	—	140	82
13	—	—	120	36
14	—	—	135	36^c^
15	—	—	135	80^d^
16	—	—	135	58^e^
17	—	—	135	63^f^
18	—	—	135	85^g^
19	—	—	135	85^h^
20	—	—	135	92^i^

^a^1 mmol **1a**, 1 mmol **2a**, 24 h. ^b^Based on **2a**. ^c^18 h. ^d^30 h. ^e^Open atmosphere. ^f^0.8 mmol **1a**. ^g^1.5 mmol **1a**. ^h^1.75 mmol **1a**. ^i^2 mmol **1a**.

Thereupon, these optimized conditions were utilized to validate the substrate scope of this direct C–C coupling reaction. A variety of aldehydes, **2a**–**r**, was reacted with 2,6-dimethylpyridine (**1a**) to obtain the corresponding products **3a**–**r** with a moderate to excellent yield (Table 2). The substrates **2a**–**d** with nitro substituents were well reacted to achieve the desired products **3a**–**d** in 68–95% yield (Table 2, entries 1–4). Distinctly, 2-nitrobenzaldehyde (**2c**) gave the expected product **3c** along with the dehydrated product **4c** (Table 2, entry 3). Substrates containing a halogen, such as fluorine, chlorine, and bromine, were efficiently reacted to afford the products **3e**–**h** in successively moderate yields (Table 2, entries 5–8). Notably, 3-fluorobenzaldehyde (**2e**) also reacted well and provided the alcohol **3e** in 55% yield along with the alkene product **4e** in 19% yield (Table 2, entry 5). Exceptionally, substrates with groups such as cyano, trifluoromethyl, and formyl were reacted and provided the respective dehydrated alkenylpyridine compounds **4i**–**k** in 77%, 71%, and 92% yield, respectively (Table 2, entries 9–11). 4-Acetamidobenzaldehyde (**2l**) also reacted but provided a low product yield of 22% **3l** (Table 2, entry 12). Starting materials with a *para*- and *ortho*-hydroxy group provided the corresponding alkenylpyridine products **4m** and **4n** in 58% and 34% yield, respectively (Table 2, entries 13 and 14). 4-Methylbenzaldehyde (**2o**) gave the respective dehydrated product **4o** in 46% yield upon reaction for 48 h (Table 2, entry 15). Simple benzaldehyde (**2p**) was also tolerated well and furnished the corresponding product **3p** in 45% yield (Table 2, entry 16). The heteroaromatic aldehyde 2-pyridinecarbaldehyde (**2q**) gave the respective dehydrated product **4q** in 96% yield, whereas 2-thiophene (**2r**) resulted in the desired product **3r** but in a low yield (Table 2, entries 17 and 18).

**Table 2:** Variation of the aldehyde component **2**.^a^

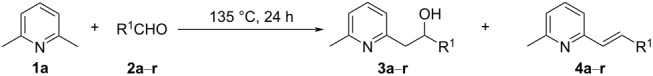					

entry	substrate **2**	product **3**	product **4**	yield (%)^b^	

				**3**	**4**

1	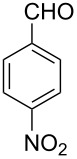 **2a**	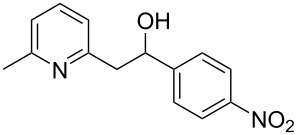 **3a**	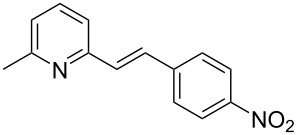 **4a**	92	0
2	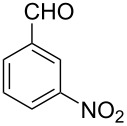 **2b**	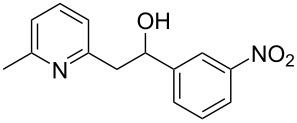 **3b**	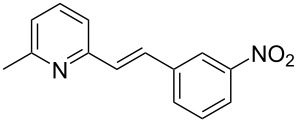 **4b**	68	0
3	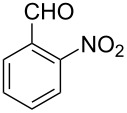 **2c**	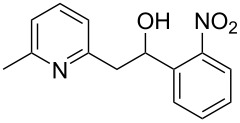 **3c**	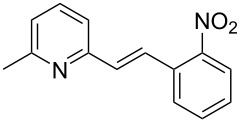 **4c**	74	17
4	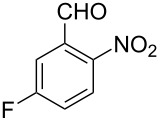 **2d**	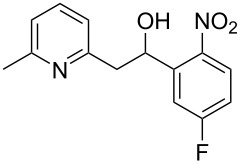 **3d**	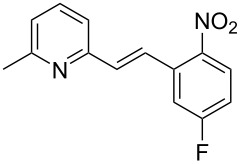 **4d**	95	0
5	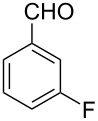 **2e**	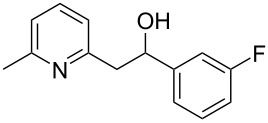 **3e**	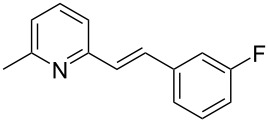 **4e**	55	19
6	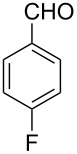 **2f**	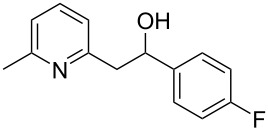 **3f**	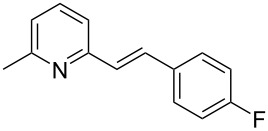 **4f**	52	0
7	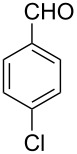 **2g**	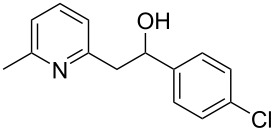 **3g**	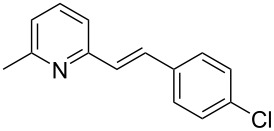 **4g**	48	0
8	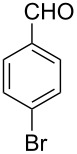 **2h**	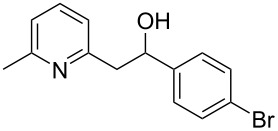 **3h**	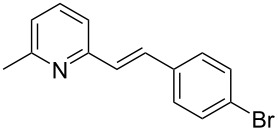 **4h**	42	0
9	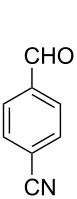 **2i**	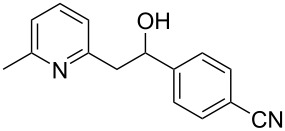 **3i**	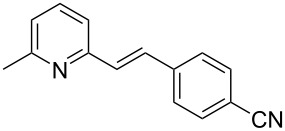 **4i**	0	77
10	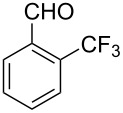 **2j**	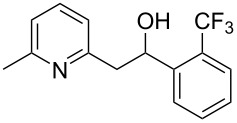 **3j**	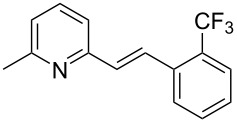 **4j**	0	71
11	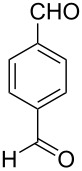 **2k**	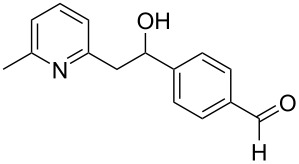 **3k**	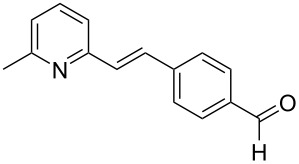 **4k**	0	92
12	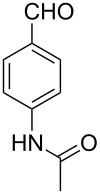 **2l**	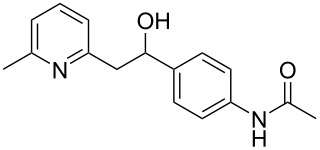 **3l**	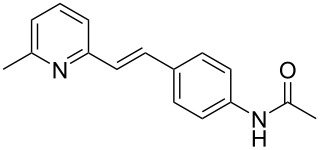 **4l**	22	0
13	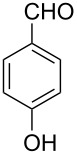 **2m**	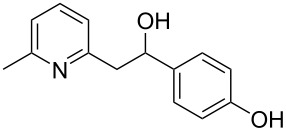 **3m**	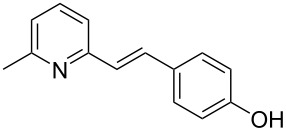 **4m**	0	58
14	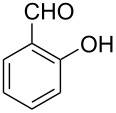 **2n**	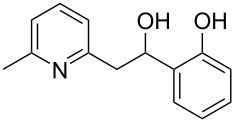 **3n**	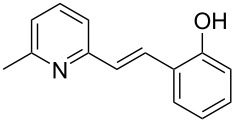 **4n**	0	34
15	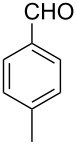 **2o**	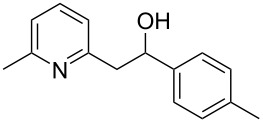 **3o**	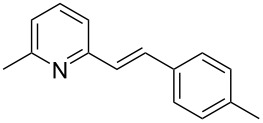 **4o**	0	46^c^
16	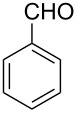 **2p**	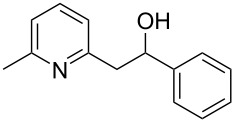 **3p**	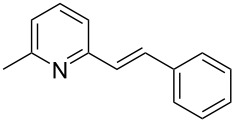 **4p**	45	0
17	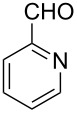 **2q**	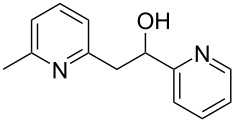 **3q**	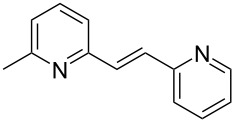 **4q**	0	96
18	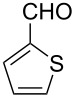 **2r**	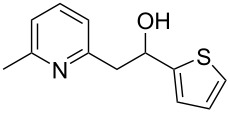 **3r**	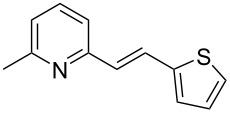 **4r**	29^c^	0

^a^2 mmol **1a**, 1 mmol **2**, 24 h. ^b^Based on **2**. ^c^48 h.

Subsequently, several azaarenes in combination with 4-nitrobenzaldehyde (**2a**) were also evaluated under optimized conditions to extend the substrate scope. Firstly 2,4,6-collidine (**1b**) was successfully reacted for 48 h to obtain the successive product **5b** in 87% yield (Table 3, entry 1). Next, 2-ethylpyridine (**1c**) was made to react under standard reaction conditions. To our surprise, 1-(4-nitrophenyl)-2-(pyridin-2-yl)propan-1-ol (**5c**) was formed in 53% yield, and the formation of this product displays the reactivity of the benzylic C–H group attached in the *ortho* position of the pyridine ring (Table 3, entry 2). The reaction of 2-methylpyridine (**1d**) with **2a** for 48 h gave the desired alcohol **5d** in 42% yield along with the corresponding dehydrated alkenylpyridine compound **6d** in 16% yield (Table 3, entry 3). Different from the 2,6-substitution pattern, 3,5-dimethylpyridine (**1e**) was also tested under standard reaction conditions upon increasing the reaction time to 48 h, which did not furnish the corresponding products (Table 3, entry 4). Later, to screen the compatibility of the azaarenes, various quinolines were screened (Table 3, entries 5–8). Among these, only 2-methylquinoline (**1f**) and 6-fluoro-2-methylquinoline (**1g**) gave the dehydrated alkenylazaarenes **6f** and **6g** as the products in 48% and 45% yield, respectively (Table 3, entries 5 and 6). We also tried this reaction with 6-chloro-2-methylquinoline (**1h**) and 6-bromo-2-methylquinoline (**1g**), but unfortunately the reactions did not proceed well, and the desired products **6h** and **6i** were not formed (Table 3, entries 7 and 8).

**Table 3:** Variation of the azaarene component **1**.^a^

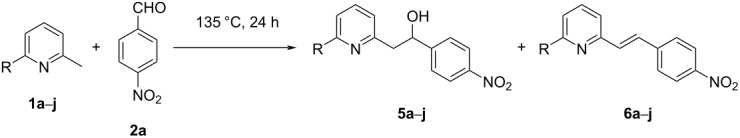					

entry	substrate	product **5**	product **6**	yield (%)^b^	

				**5**	**6**

1	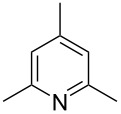 **1b**	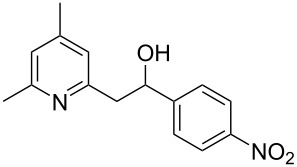 **5b**	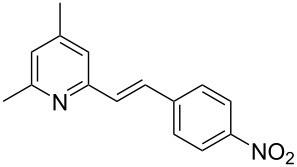 **6b**	87^c^	0
2	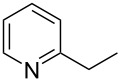 **1c**	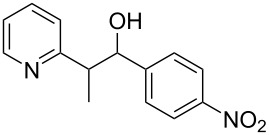 **5c**	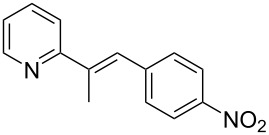 **6c**	53^c^	0
3	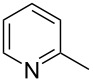 **1d**	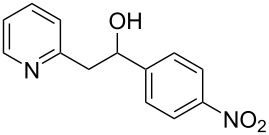 **5d**	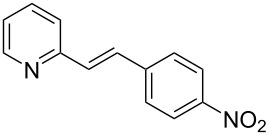 **6d**	42^c^	16^c^
4	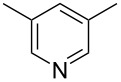 **1e**	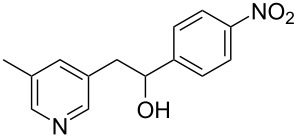 **5e**	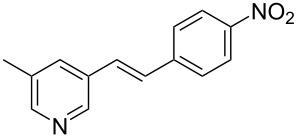 **6e**	0	0
5	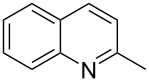 **1f**	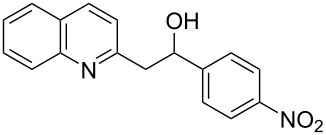 **5f**	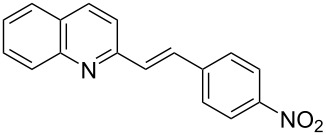 **6f**	0	48
6	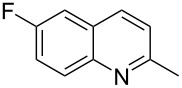 **1g**	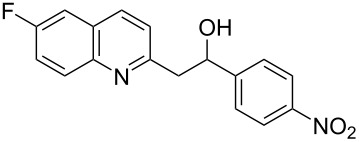 **5g**	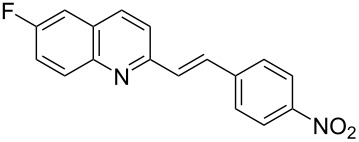 **6g**	0	45
7	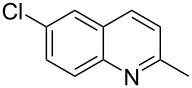 **1h**	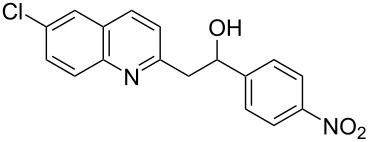 **5h**	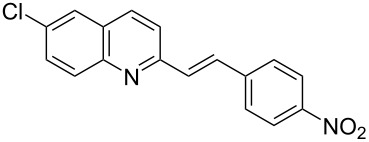 **6h**	0	0
8	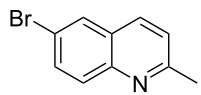 **1i**	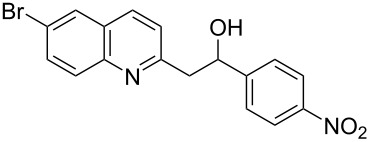 **5i**	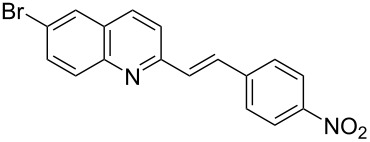 **6i**	0	0

^a^2 mmol **1**, 1 mmol **2a**, 24 h. ^b^Based on **2a**. ^c^48 h.

**Scheme 4:**
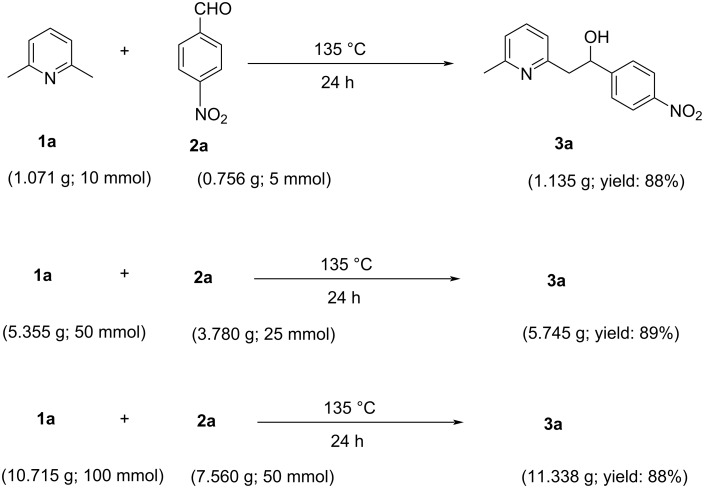
Large-scale experiments for the synthesis of 2-(6-methylpyridin-2-yl)-1-(4-nitrophenyl)ethan-1-ol (**3a**) from 2,6-dimethylpyridine (**1a**) and 4-nitrobenzaldehyde (**2a**).

However, some dehydrated alkenyl products were obtained, which may possess wide biological activities of great demand. This reaction was also carried out on a gram scale, and the results display the potential for large-scale applications as the yields were not drastically changed when shifting from a mmol scale to a gram scale (1 g, 5 g, and 10 g). These results are shown in Scheme 4.

**Scheme 5:**
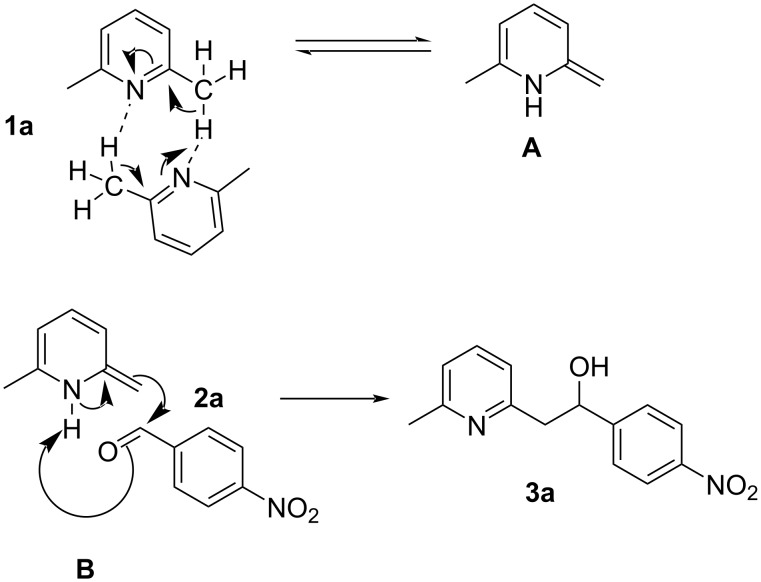
Plausible mechanism for the formation of 2-(6-methylpyridin-2-yl)-1-(4-nitrophenyl)ethan-1-ol (**3a**) under standard reaction conditions from 2,6-dimethylpyridine (**1a**) and 4-nitrobenzaldehyde (**2a**).

Based on the experimental investigations and literature reports [46], conclusions were drawn, and a plausible mechanism was suggested. Under standard reaction conditions, 2 mmol of 2,6-dimethylpyridine participates in inter reaction (1 mmol of **1a** will inter react to another 1 mmol of **1a**) and this inter reaction influences the nitrogen atom present in the compound to act as a Brønsted base. This resulting Brønsted base accepts benzylic C–H protons to furnish the respective enamine **A**, which facilitates the further nucleophilic addition to 4-nitrobenzaldehyde (**2a**). The product of this is the intermediate **B**, which favors the formation of the corresponding desired product **3a**, as shown in Scheme 5.

## Conclusion

In summary, this work provides a green and simple synthetic methodology for the synthesis of higher azaarenes from pyridines and quinolines with aromatic aldehydes, avoiding the use of solvents and a catalyst. Despite of previous reports under catalyst- and solvent-free conditions, this approach features specific traits, such as good yields and simple reaction conditions. This method requires no reagent purification and is a new and clean process. A variety of aromatic aldehydes, including electron-donating, electron-withdrawing, as well as halogen groups was screened, affording the products in moderate to excellent yields. Azaarenes such as pyridines with various substitution patterns and a few quinolines were also reacted with moderate to excellent yields. This draws the conclusion that the benzylic C–H unit of 2-alkylpyridine/quinolines actively participates in this addition reaction. However, in some cases, 2-alkenyl compounds were formed. Nevertheless, these are of equivalent significance. Moreover, this method has a high atom economy and reduces large amounts of waste generated by the unnecessary utilization of catalysts and solvents. Notably, this reaction is compatible with a gram scale, and further research is yet to be developed towards more sustainability.

## Supporting Information

File 1 Experimental procedures, compound characterization data, and NMR spectra.
