# Supplementary material for: An atom-economical addition of methyl azaarenes with aromatic aldehydes via benzylic C(sp3)–H bond functionalization under solvent- and catalyst-free conditions
Source: Beilstein J Org Chem. 2020 Dec 23;16:3093–103. doi: 10.3762/bjoc.16.259 (PMC7770386; doi:10.3762/bjoc.16.259)
Supplement: File 1 — Experimental procedures, compound characterization data, and NMR spectra. [file Beilstein_J_Org_Chem-16-3093-s001.pdf]

BEILSTEIN JOURNAL OF ORGANIC CHEMISTRY

Supporting Information

for

An atom-economical addition of methyl azaarenes with aromatic aldehydes via benzylic C(sp^3)–H bond functionalization under solvent- and catalyst-free conditions

Divya Rohini Yennamaneni, Vasu Amrutham, Krishna Sai Gajula, Rammurthy Banothu, Murali Boosa and Narender Nama

Beilstein J. Org. Chem. **2020,** *16,* 3093–3103. doi:10.3762/bjoc.16.259

Experimental procedures, compound characterization data, and NMR spectra

License and Terms: This is a supporting information file under the terms of the Creative Commons Attribution License (https://creativecommons.org/licenses/by/4.0). Please note that the reuse, redistribution and reproduction in particular requires that the author(s) and source are credited and that individual graphics may be subject to special legal provisions.
The license is subject to the *Beilstein Journal of Organic Chemistry* terms and conditions: (https://www.beilstein-journals.org/bjoc/terms)

Table of Contents

1. Experimental section……………………………………………..…………S1
   i) Chemicals
   ii) General information
   iii) General procedure

2. Spectroscopic data of all products ………………………………….……....S3

3. Copies of ^{1}H and ^{13}C NMR spectra of all products….…………………….………S11

4. References………………..……………………................………...................S37

1. Experimental section

i) Chemicals

Ammonium β zeolite ($NH_4\beta$) (Si/Al = 19) was purchased from Alfa Aesar, England and calcined at 500 °C for 10 h to afford Hβ. MCM-41 (Si/Al = 30) was synthesized [1], and H-mordenite (Si/Al = 12.5) was purchased from Zeolyst C. V. Azaarenes and benzaldehyde derivatives were purchased from Sigma-Aldrich. All remaining chemicals used in this study were used as received.

ii) General information

^{1}HNMR spectra were recorded at 300, 400, or 500 MHz and ^{13}CNMR spectra at 75, 100, or 125 MHz in $CDCl_3$. The chemical shifts (δ) are described in ppm relative to TMS as an internal standard for ^{1}HNMR and $CDCl_3$ for ^{13}CNMR spectra. Coupling constants (*J*) are reported in Hz, and multiplicities are indicated as follows: s (singlet), br s (broad singlet), d (doublet), dd (doublet of doublet), t (triplet), m (multiplet). The mass spectrometric analysis was performed using a high-resolution Q-TOF mass spectrometer. The GC analysis was carried out using a GC Shimadzu (GC-2014) gas chromatograph equipped with an FID detector and a capillary column (EB-5, length 30 m, inner diameter 0.25 mm, film 0.25 mm). TLC inspections were

carried out on silica gel 60 F_{254} plates. Column chromatography was performed on silica gel (100–200 mesh) using *n*-hexane/EtOAc as an eluent.

iii) General procedure

Reactions were performed in a magnetically stirred 15 mL sealed vial and placed in a temperature-controlled oil bath. The appropriate benzaldehyde (1 mmol) was added to a pyridine or quinoline compounds (2 mmol), and the reaction mixture was allowed to stir at 135 °C for 24 hours. After the disappearance of the starting materials (as monitored by TLC analysis) or after the appropriate time, the reaction mixture was cooled to room temperature. The crude residue was further purified by column chromatography using silica gel (100–200 mesh) to afford pure products. All the products were identified on the basis of NMR and mass spectral data.

2. Spectroscopic data of all products

2-(6-Methylpyridin-2-yl)-1-(4-nitrophenyl)ethan-1-ol (3a)[2]

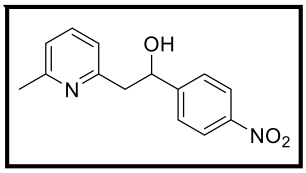

^{1}H NMR (300 MHz, $CDCl_3$) δ (ppm) = 8.20 (d, J = 8.5 Hz, 2H), 7.60 (d, J = 8.5 Hz, 2H), 7.52 (t, J = 7.7 Hz, 1H), 7.07 (d, J = 7.7 Hz, 1H), 6.90 (d, J = 7.4 Hz, 1H), 5.25 (dd, J = 3.0, 8.5 Hz, 1H), 3.15-2.99 (m, 2H), 2.57 (s, 3H); ^{13}C NMR (100 MHz, $CDCl_3$) δ (ppm) = 158.12, 157.42, 151.62, 147.03, 137.41, 126.54, 123.50, 121.61, 120.59, 72.50, 44.46, 24.25.

2-(6-Methylpyridin-2-yl)-1-(3-nitrophenyl)ethan-1-ol (3b)[2]

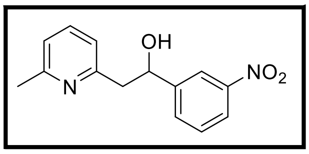

^{1}H NMR (400 MHz, $CDCl_3$) δ (ppm) = 8.30 (s, 1H), 8.13-8.10 (m, 1H), 7.79 (d, J = 8.3 Hz, 1H), 7.53 (qt, J = 7.6 Hz, 2H), 7.07 (d, J = 7.7 Hz, 1H), 6.91 (d, J = 7.6 Hz, 1H), 5.24 (dd, J = 3.2, 8.6 Hz, 1H), 3.15-3.02 (m, 2H), 2.57 (s, 3H); ^{13}C NMR (125 MHz, $CDCl_3$) δ (ppm) = 158.22, 157.47, 148.24, 146.42, 137.40, 131.96, 129.18, 122.14, 121.60, 120.95, 120.59, 72.39, 44.56, 24.31.

2-(6-Methylpyridin-2-yl)-1-(2-nitrophenyl)ethan-1-ol (3c)[2]

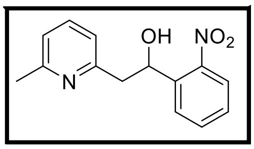

^{1}H NMR (500 MHz, $CDCl_3$) δ (ppm) = 7.97 (dd, J = 7.9, 16.9 Hz, 2H), 7.64 (t, J = 7.6 Hz, 1H), 7.54 (t, J = 7.6 Hz, 1H), 7.41 (t, J = 7.6 Hz, 1H), 7.07 (d, J = 7.7 Hz, 1H), 6.98 (d, J = 7.6 Hz, 1H), 6.89 (brs, 1H), 5.63 (dd, J = 2.1, 9.0 Hz, 1H), 3.30 (dd, J = 2.1, 14.8 Hz, 1H), 3.02-2.97 (m, 1H), 2.57 (s, 3H); ^{13}C NMR (75 MHz, $CDCl_3$) δ (ppm) = 158.70, 157.31, 147.53, 139.67, 137.45, 133.43, 128.55, 127.83, 124.17, 121.52, 120.60, 69.16, 44.19, 24.32.

(*E*)-2-Methyl-6-(2-nitrostyryl)pyridine (4c)

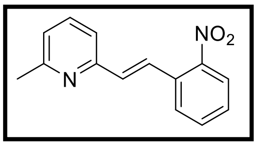

^{1}H NMR (400 MHz, $CDCl_3$) *δ* (ppm) = 7.99-7.95 (m, 2H), 7.81-7.79 (m, 1H), 7.62-7.56 (m, 2H), 7.45-7.40 (m, 1H), 7.32 (d, *J* = 7.7 Hz, 1H), 7.16 (d, *J* = 16.0 Hz, 1H), 7.06 (d, *J* = 7.5 Hz, 1H), 2.58 (s, 3H); ^{13}C NMR (100 MHz, $CDCl_3$) *δ* (ppm) = 158.49, 154.28, 148.24, 136.74, 133.65, 133.09, 132.54, 128.42, 127.27, 124.72, 122.50, 118.93, 24.57; HRMS (ESI): m/z calculated for $C_{14}H_{13}O_2N_2[M+H]^+$, 241.09715, found: 241.09674.

1-(5-Fluoro-2-nitrophenyl)-2-(6-methylpyridin-2-yl)ethan-1-ol (3d)

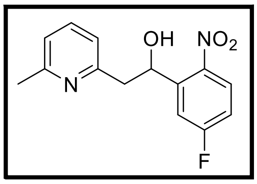

^{1}H NMR (400 MHz, $CDCl_3$) *δ* (ppm) = 8.08 (dd, *J* = 5.0, 9.0 Hz, 1H), 7.72 (dd, *J* = 2.8, 9.7 Hz, 1H), 7.55 (t, *J* = 7.7 Hz, 1H), 7.11-7.06 (m, 2H), 6.98 (d, *J* = 7.7 Hz, 1H), 5.68 (d, *J* = 9.7 Hz, 1H), 3.30 (dd, *J* = 2.2, 14.9 Hz, 1H), 2.94 (dd, *J* = 8.9, 14.9 Hz, 1H), 2.57 (s, 3H); ^{13}C NMR (100 MHz, $CDCl_3$) *δ* (ppm) = 166.74, 164.18, 158.37, 157.34, 144.15 (d, *J* = 8.7 Hz), 143.20, 137.56, 127.40 (d, *J* = 9.8 Hz), 121.67, 120.57, 115.7 (d, *J* = 25.0 Hz), 114.90 (d, *J* = 23.9 Hz), 69.13, 43.76, 24.29; HRMS (ESI): m/z calculated for $C_{14}H_{14}O_3N_2F[M+H]^+$, 277.0983, found: 277.0978.

1-(3-Fluorophenyl)-2-(6-methylpyridin-2-yl)ethan-1-ol (3e)[2]

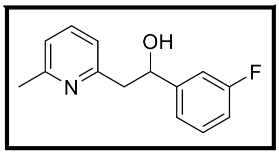

^{1}H NMR (400 MHz, $CDCl_3$) *δ* (ppm) = 7.51 (t, *J* = 7.7 Hz, 1H), 7.32-7.26 (m, 1H), 7.18-7.15 (m, 2H), 7.04 (d, *J* = 7.8 Hz, 1H), 6.96-6.89 (m, 2H), 5.13 (dd, *J* = 4.6, 7.4 Hz, 1H), 3.06-3.04 (m, 2H), 2.56 (s, 3H); ^{13}C NMR (75 MHz, $CDCl_3$) *δ* (ppm) = 162.95 (d, *J* = 245.7 Hz), 158.72, 157.37, 146.94 (d, *J* = 7.2 Hz), 137.23, 129.67 (d, *J* = 8.03 Hz), 121.37, 120.56, 114.04, 113.76, 112.93, 112.64, 72.71, 44.95, 24.32.

(E)-2-(3-Fluorostyryl)-6-methylpyridine (4e)[2]

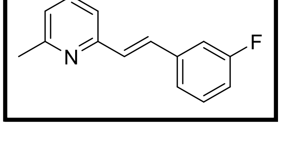

^{1}H NMR (400 MHz, $CDCl_3$) *δ* (ppm) = 7.58-7.53 (m, 2H), 7.35-7.28 (m, 3H), 7.22 (d, *J* = 7.5 Hz, 1H), 7.15 (d, *J* = 16.1 Hz, 1H), 7.04 (d, *J* = 7.7 Hz, 1H), 7.00-6.95 (m,1H), 2.59 (s, 3H); ^{13}C NMR (75 MHz, $CDCl_3$) *δ* (ppm) = 163.09 (d, *J* = 244.9 Hz), 158.36, 154.51, 139.14 (d, *J*

= 8.0 Hz), 136.69, 131.07, 130.05 (d, J = 8.8 Hz), 129.59, 122.96, 122.01, 119.12, 114.90 (d, J = 21.6 Hz), 113.25 (d, J = 21.6 Hz), 24.61.

1-(4-Fluorophenyl)-2-(6-methylpyridin-2-yl)ethan-1-ol (3f)[2]

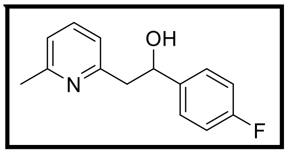

^{1}H NMR (500 MHz, $CDCl_3$) δ (ppm) = 7.50 (t, J = 7.6 Hz, 1H), 7.40-7.37 (m, 2H), 7.05-7.00 (m, 3H), 6.89 (d, J = 7.6 Hz, 1H), 5.11 (dd, J = 3.9, 8.0 Hz, 1H), 3.08-3.00 (m, 2H), 2.55 (s, 3H); ^{13}C NMR (75 MHz, $CDCl_3$) δ (ppm) = 161.95 (d, J = 244.2 Hz), 158.84, 157.36, 139.91 (d, J = 2.7 Hz), 137.21, 127.37 (d, J = 8.2 Hz), 121.33, 120.58, 115.13, 114.86, 72.73, 45.26, 24.29.

1-(4-Chlorophenyl)-2-(6-methylpyridin-2-yl)ethan-1-ol (3g)[2]

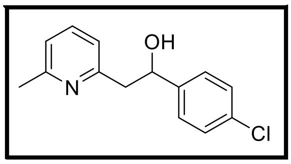

^{1}H NMR (400 MHz, $CDCl_3$) δ (ppm) = 7.51 (t, J = 7.7 Hz, 1H), 7.37-7.29 (m, 4H), 7.04 (d, J = 7.7 Hz, 1H), 6.89 (d, J = 7.5 Hz, 1H), 5.11 (t, J = 6.1 Hz, 1H), 3.03 (d, J = 5.8 Hz, 2H), 2.55 (s, 3H); ^{13}C NMR (125 MHz, $CDCl_3$) δ (ppm) = 158.76, 157.38, 142.70, 137.24, 132.73, 128.36, 127.19, 121.37, 120.57, 72.69, 45.04, 24.33.

1-(4-Bromophenyl)-2-(6-methylpyridin-2-yl)ethan-1-ol (3h)

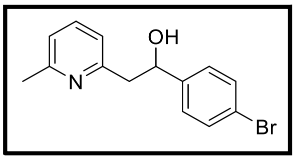

^{1}H NMR (400 MHz, $CDCl_3$) δ (ppm) = 7.51 (t, J = 7.6 Hz, 1H), 7.47-7.44 (m, 2H), 7.32-7.29 (m, 2H), 7.05 (d, J = 7.6 Hz, 1H), 6.89 (d, J = 7.6 Hz, 1H), 5.09 (t, J = 5.9 Hz, 1H), 3.06-2.98 (m, 2H), 2.55 (s, 3H); ^{13}C NMR (125 MHz, $CDCl_3$) δ (ppm) = 158.73, 157.38, 143.23, 137.24, 131.30, 127.56, 121.37, 120.57, 72.72, 44.98, 24.33; HRMS (ESI): m/z calculated for $C_{14}H_{15}$ ONBr$[M+H]^{+}$, 292.03315, found: 292.03254.

(*E*)-4-(2-(6-Methylpyridin-2-yl)vinyl)benzonitrile (4i)[2]

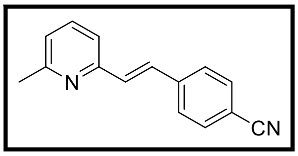

^{1}H NMR (500 MHz, $CDCl_3$) δ (ppm) = 7.64 (s, 4H), 7.62-7.57 (m, 2H), 7.26-7.22 (m, 2H), 7.07 (d, J = 7.6 Hz, 1H), 2.59 (s, 3H); ^{13}C NMR (75 MHz, $CDCl_3$) δ (ppm) = 158.55, 153.84, 141.25, 136.79, 132.39, 131.72, 130.19, 127.31, 122.56, 119.66, 118.86, 111.02, 24.58.

(*E*)-2-Methyl-6-(2-(trifluoromethyl)styryl)pyridine (4j)[2]

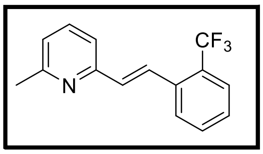

^{1}H NMR (400 MHz, $CDCl_3$) δ (ppm) = 7.89-7.82 (m, 2H), 7.68 (d, J = 7.9 Hz, 1H), 7.59-7.52 (m, 2H), 7.37 (t, J = 7.6 Hz, 1H), 7.33 (d, J = 7.6 Hz, 1H), 7.16 (d, J = 15.8 Hz, 1H), 7.05 (d, J = 7.6 Hz, 1H), 2.59 (s, 3H); ^{13}C NMR (100 MHz, $CDCl_3$) δ (ppm) = 158.40, 154.73, 136.67, 135.89, 132.78, 131.89, 128.04, 127.72, 127.65, 127.30, 125.99-125.72 (m), 122.99, 122.18, 118.52, 24.57.

(*E*)-4-(2-(6-Methylpyridin-2-yl)vinyl)benzaldehyde (4k)

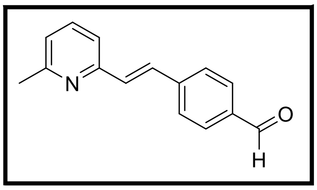

^{1}H NMR (400 MHz, $CDCl_3$) δ (ppm) = 10.00 (s, 1H), 7.89-7.87 (m, 2H), 7.72-7.70 (m, 2H), 7.67-7.63 (m, 1H), 7.58 (t, J = 7.7 Hz, 1H), 7.31-7.24 (m, 2H), 7.07 (d, J = 7.5 Hz, 1H), 2.60 (s, 3H); ^{13}C NMR (100 MHz, $CDCl_3$) δ (ppm) = 191.54, 158.53, 154.15, 142.83, 136.76, 135.64, 131.58, 130.85, 130.13, 127.41, 122.42, 119.55, 24.60; HRMS (ESI): m/z calculated for $C_{15}H_{14}ON[M+H]^+$, 224.10699, found: 224.10648.

***N*-(4-(1-Hydroxy-2-(6-methylpyridin-2-yl)ethyl)phenyl)acetamide (3l)**

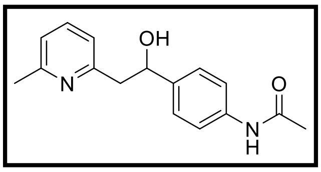

^{1}H NMR (500 MHz, $CDCl_3$+DMSO-d_6) δ (ppm) = 7.51 (t, J = 7.7 Hz, 1H), 7.46-7.35 (m, 4H), 7.04 (d, J = 7.7 Hz, 1H), 6.90 (d, J = 7.7 Hz, 1H), 5.09 (dd, J = 4.0, 8.1 Hz, 1H), 3.11-3.01 (m, 2H), 2.55 (s, 3H), 2.16 (s, 3H); ^{13}C NMR (75 MHz, $CDCl_3$+DMSO-d_6) δ (ppm) = 168.03, 157.97, 156.28, 138.90, 137.14, 136.12, 125.34, 120.25, 120.04, 118.66, 72.02, 45.26, 39.47, 23.50, 23.39; HRMS (ESI): m/z calculated for $C_{16}H_{19}O_2N_2$ $[M+H]^+$, 271.1441 found: 271.1434.

(*E*)-4-(2-(6-Methylpyridin-2-yl)vinyl)phenol (4m)

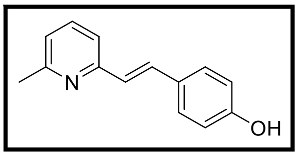

^{1}H NMR (400 MHz, $CDCl_3$+DMSO-d_6) δ (ppm) = 7.62 (t, J = 7.7 Hz, 1H), 7.54-7.46 (m, 3H), 7.27 (d, J = 7.7 Hz, 1H), 7.06-7.01 (m, 2H), 6.78 (d, J = 8.5 Hz, 2H), 2.47 (s, 3H); ^{13}C NMR (75 MHz, $CDCl_3$+DMSO-d_6) δ (ppm) = 157.19, 154.76, 136.03, 131.61, 127.71, 127.30, 124.54, 120.50, 117.62, 115.17, 23.85; HRMS (ESI): m/z calculated for $C_{14}H_{14}ON[M+H]^+$, 212.1070, found: 212.1066.

(*E*)-2-(2-(6-Methylpyridin-2-yl)vinyl)phenol (4n)

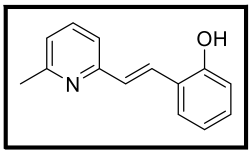

^{1}H NMR (400 MHz, $CDCl_3$+DMSO-d_6) δ (ppm) = 7.88 (d, J = 16.2 Hz, 1H), 7.65-7.53 (m, 2H), 7.28-7.24 (m, 1H), 7.14-6.76 (m, 5H), 2.49 (s, 3H); ^{13}C NMR (75 MHz, $CDCl_3$+DMSO-d_6) δ (ppm) = 157.48, 155.39, 154.78, 136.85, 129.11, 127.32, 126.85, 123.13, 121.29, 119.21, 115.80, 24.19; HRMS (ESI): m/z calculated for $C_{14}H_{16}O_2N[M+H+H_2O]^+$, 230.1176, found: 230.1170.

(*E*)-2-Methyl-6-(4-methylstyryl)pyridine (4o)[2]

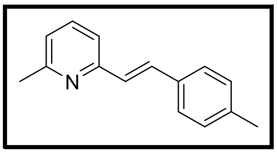

^{1}H NMR (400 MHz, $CDCl_3$) δ (ppm) = 7.56-7.51 (m, 2H), 7.48-7.46 (m, 2H), 7.22 (d, J = 7.7 Hz, 1H), 7.18-7.10 (m, 3H), 7.00 (d, J = 7.5 Hz, 1H), 2.58 (s, 3H), 2.36 (s, 3H) ; ^{13}C NMR (100 MHz, $CDCl_3$) δ (ppm) = 158.19, 155.31, 138.14, 136.58, 134.00, 132.30, 129.37, 127.46, 126.97, 121.46, 118.58, 24.64, 21.28.

2-(6-Methylpyridin-2-yl)-1-phenylethan-1-ol (3p)[2]

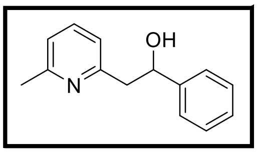

^{1}H NMR (400 MHz, $CDCl_3$) δ (ppm) = 7.52 (t, J = 7.5 Hz, 1H), 7.44-7.41 (m, 2H), 7.35-7.31 (m, 2H), 7.27-7.23 (m, 1H), 7.05 (d, J = 7.7 Hz, 1H), 6.91 (d, J = 7.5 Hz, 1H), 5.16-5.12 (m,

1H), 3.13-3.11 (m, 2H), 2.57 (s, 3H) ; ^{13}C NMR (75 MHz, $CDCl_3$) δ (ppm) = 158.72, 157.17, 144.02, 137.42, 132.90, 129.95, 128.23, 127.17, 125.77, 121.46, 120.86, 73.44, 45.28, 23.94.

(*E*)-2-Methyl-6-(2-(pyridin-2-yl)vinyl)pyridine (4q)

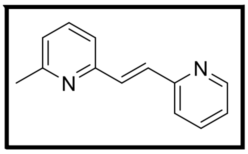

^{1}H NMR (400 MHz, $CDCl_3$) δ (ppm) = 8.62 (d, J = 4.7 Hz, 1H), 7.71-7.62 (m, 3H), 7.56 (t, J = 7.7 Hz, 1H), 7.46 (d, J = 7.8 Hz, 1H), 7.26 (d, J = 6.8 Hz, 1H), 7.17 (dd, J = 1.1, 4.8 Hz, 1H), 7.05 (d, J = 7.7 Hz, 1H), 2.59 (s, 3H); ^{13}C NMR (100 MHz, $CDCl_3$) δ (ppm) = 158.37, 155.19, 154.33, 149.64, 136.73, 136.53, 132.09, 131.45, 122.88, 122.44, 122.32, 120.16, 24.63; HRMS (ESI): m/z calculated for $C_{13}H_{13}N_2$[M+H]$^+$,197.1073, found: 197.1071.

2-(6-Methylpyridin-2-yl)-1-(thiophen-2-yl)ethan-1-ol (3r)

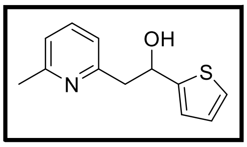

^{1}H NMR (400 MHz, $CDCl_3$) δ (ppm) = 7.51 (t, J = 7.7 Hz, 1H), 7.21 (dd, J = 1.3, 4.8 Hz, 1H), 7.04 (d, J = 7.7 Hz, 1H), 6.98-6.93 (m, 3H), 5.40 (dt, J = 0.7, 5.9 Hz, 1H), 3.20 (d, J = 5.7 Hz, 2H), 2.54 (s, 3H); ^{13}C NMR (100 MHz, $CDCl_3$) δ (ppm) = 158.59, 157.34, 148.36, 137.18, 126.43, 124.06, 122.88, 121.35, 120.68, 69.64, 44.98, 24.27; HRMS (ESI): m/z calculated for $C_{12}H_{14}ONS$[M+H]$^+$, 220.07906, found: 220.07861.

2-(4,6-Dimethylpyridin-2-yl)-1-(4-nitrophenyl)ethan-1-ol (5b)[3]

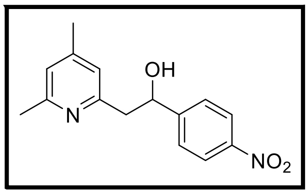

^{1}H NMR (400 MHz, $CDCl_3$) δ (ppm) = 8.19 (d, J = 8.6 Hz, 2H), 7.59 (d, J = 8.4 Hz, 2H), 6.89 (s, 1H), 6.72 (s, 1H), 5.22 (dd, J = 3.0, 8.8 Hz, 1H), 3.08-2.94 (m, 2H), 2.51 (s, 3H), 2.27 (s, 3H); ^{13}C NMR (100 MHz, $CDCl_3$) δ (ppm) = 157.91, 157.09, 151.80, 148.67, 147.03, 126.56, 123.53, 122.57, 121.56, 72.59, 44.31, 24.06, 20.90.

1-(4-Nitrophenyl)-2-(pyridin-2-yl)propan-1-ol (5c)

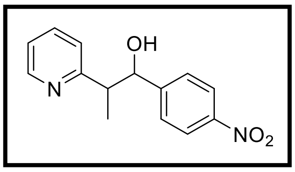

^{1}H NMR (400 MHz, $CDCl_3$) δ (ppm) = 8.52-8.50 (m, 1H), 8.09-8.06 (m, 2H), 7.53 (dt, *J* = 1.9, 7.7 Hz, 1H), 7.40-7.36 (m, 2H), 7.17-7.13 (m, 1H), 6.90 (d, *J* = 8.5 Hz, 1H), 5.04 (d, *J* = 4.5 Hz, 1H), 3.22-3.15 (m, 1H), 1.47 (d, *J* = 7.2 Hz, 3H); ^{13}C NMR (100 MHz, $CDCl_3$) δ (ppm) = 162.79, 151.94, 148.29, 146.84, 137.20, 126.74, 123.21, 122.00, 77.51, 46.64, 19.31; HRMS (ESI): m/z calculated for $C_{14}H_{15}O_3N_2[M+H]^+$, 259.10772, found: 259.10724.

1-(4-Nitrophenyl)-2-(pyridin-2-yl)ethan-1-ol (5d)[3]

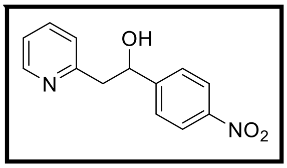

^{1}H NMR (400 MHz, $CDCl_3$) δ (ppm) = 8.55-8.53 (m, 1H), 8.21-8.17 (m, 2H), 7.65 (dt, *J* = 1.8, 7.7 Hz, 1H), 7.59 (d, *J* = 8.3 Hz, 2H), 7.25-7.22 (m, 1H), 7.10 (d, *J* = 7.8 Hz, 1H), 5.29 (dd, *J* = 3.3, 8.6 Hz, 1H), 3.22-3.08 (m, 2H); ^{13}C NMR (100 MHz, $CDCl_3$) δ (ppm) = 158.66, 151.39, 148.33, 137.32, 126.57, 123.91, 123.57, 122.14, 72.50, 44.77.

(*E*)-2-(4-Nitrostyryl)pyridine (6d)

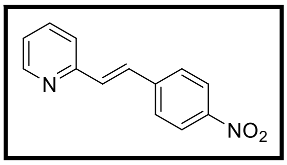

^{1}H NMR (500 MHz, $CDCl_3$) δ (ppm) = 8.64 (d, *J* = 5.6 Hz, 1H), 8.24 (d, *J* = 8.8 Hz, 2H); 7.73-7.69 (m, 4H), 7.42 (d, *J* = 7.7 Hz, 1H), 7.30 (d, *J* = 16.0 Hz, 1H), 7.24-7.21 (m, 1H); ^{13}C NMR (100 MHz, $CDCl_3$) δ (ppm) = 154.34, 149.92, 143.10, 136.78, 132.10, 130.22, 127.50, 124.13, 123.05; HRMS (ESI): m/z calculated for $C_{13}H_{11}O_2N_2$ $[M+H]^+$, 227.08150, found: 227.08111.

(*E*)-2-(4-Nitrostyryl)quinoline (6f)[3]

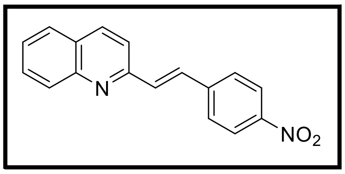

^{1}H NMR (400 MHz, $CDCl_3$) δ (ppm) = 8.26 (d, *J* = 8.8 Hz, 2H), 8.18 (d, *J* = 8.4 Hz, 1H), 8.10 (d, *J* = 8.4 Hz, 1H), 7.83-7.72 (m, 5H), 7.67 (d, *J* = 8.5 Hz, 1H), 7.56-7.50 (m, 2H); ^{13}C NMR

(100 MHz, $CDCl_3$) δ (ppm) = 154.61, 148.24, 147.36, 142.92, 136.67, 133.11, 131.65, 130.04, 129.35, 127.62, 127.55, 126.76, 124.15, 119.70.

(*E*)-6-Fluoro-2-(4-nitrostyryl)quinoline (6g)

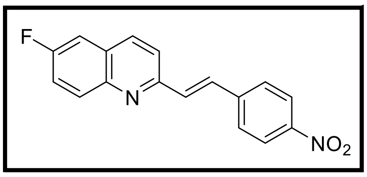

^1H NMR (400 MHz, $CDCl_3$) δ (ppm) = 8.27 (d, J = 8.8 Hz, 2H), 8.14-8.08 (m, 2H), 7.78-7.74 (m, 3H), 7.68 (d, J = 8.5 Hz, 1H), 7.54-7.42 (m, 3H); ^{13}C NMR (100 MHz, $CDCl_3$) δ (ppm) = 154.04, 147.42, 145.37, 142.85, 136.03 (d, J = 5.5 Hz), 132.77, 131.87 (d, J = 8.8 Hz), 131.66, 128.21 (d, J = 9.9 Hz), 127.65, 124.19, 120.51, 120.12, 110.68 (d, J = 21.4 Hz) ; HRMS (ESI): m/z calculated for $C_{17}H_{12}O_2N_2F[M+H]^+$, 295.0877, found: 295.0873.

3. Copies of ^{1}H and ^{13}C NMR spectra of all products

2-(6-Methylpyridin-2-yl)-1-(4-nitrophenyl)ethan-1-ol (3a)

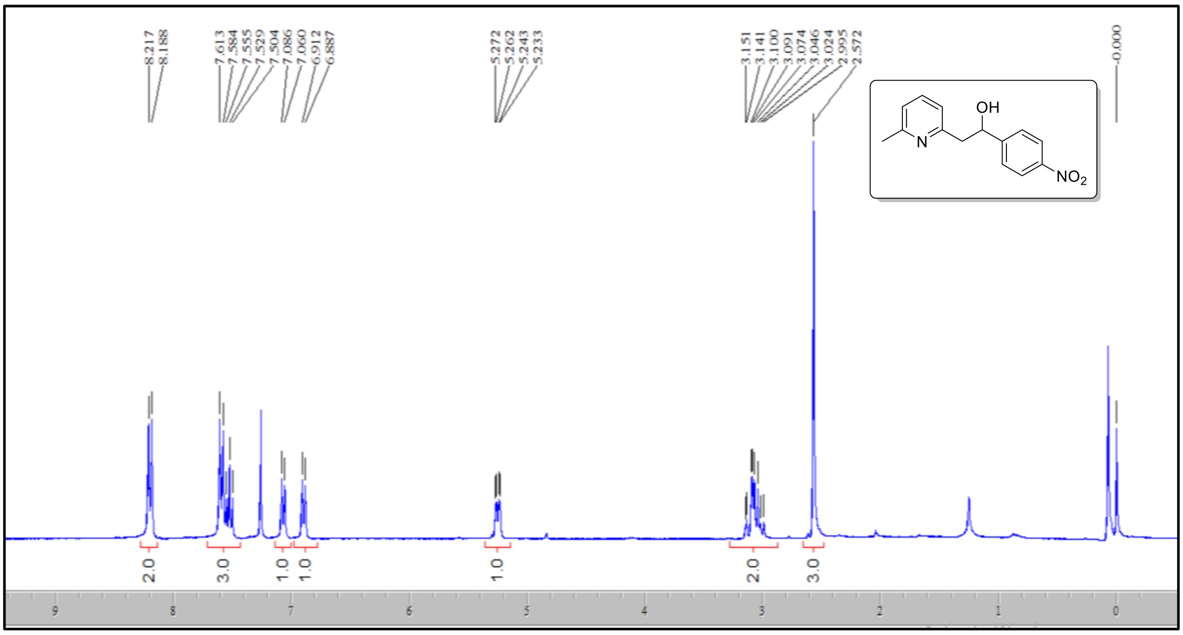

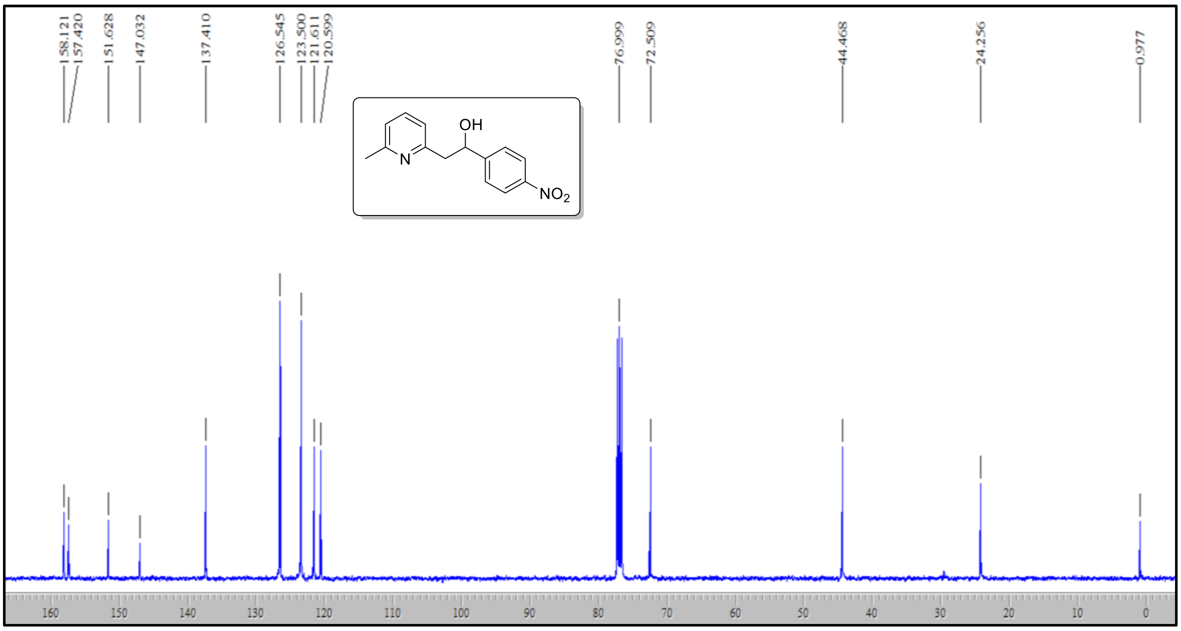

2-(6-Methylpyridin-2-yl)-1-(3-nitrophenyl)ethan-1-ol (3b)

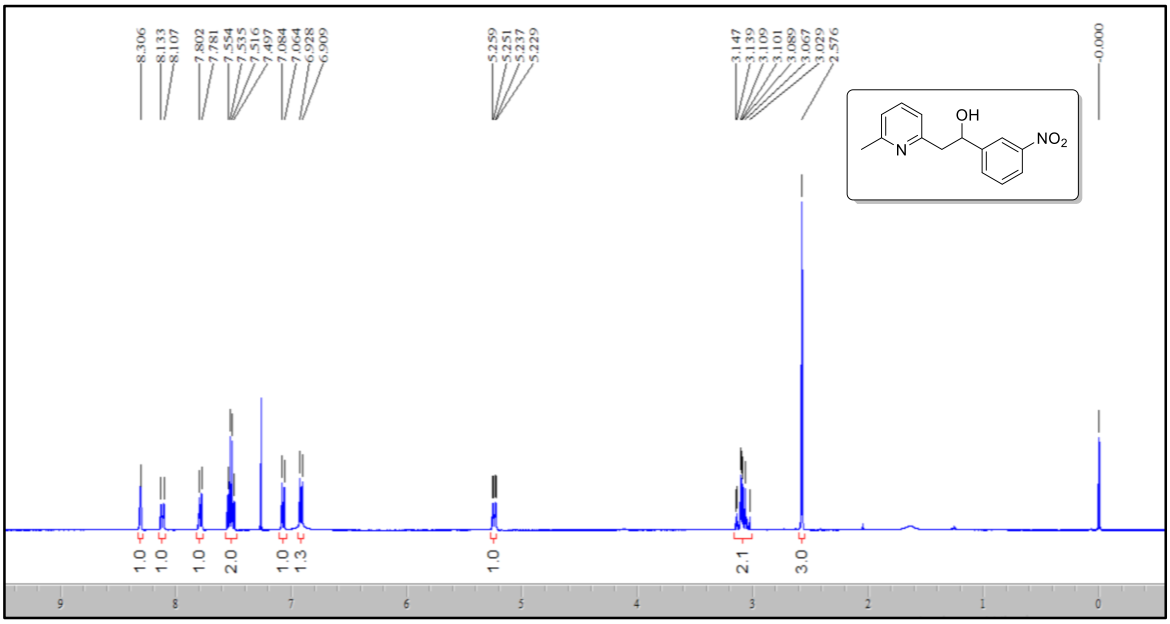

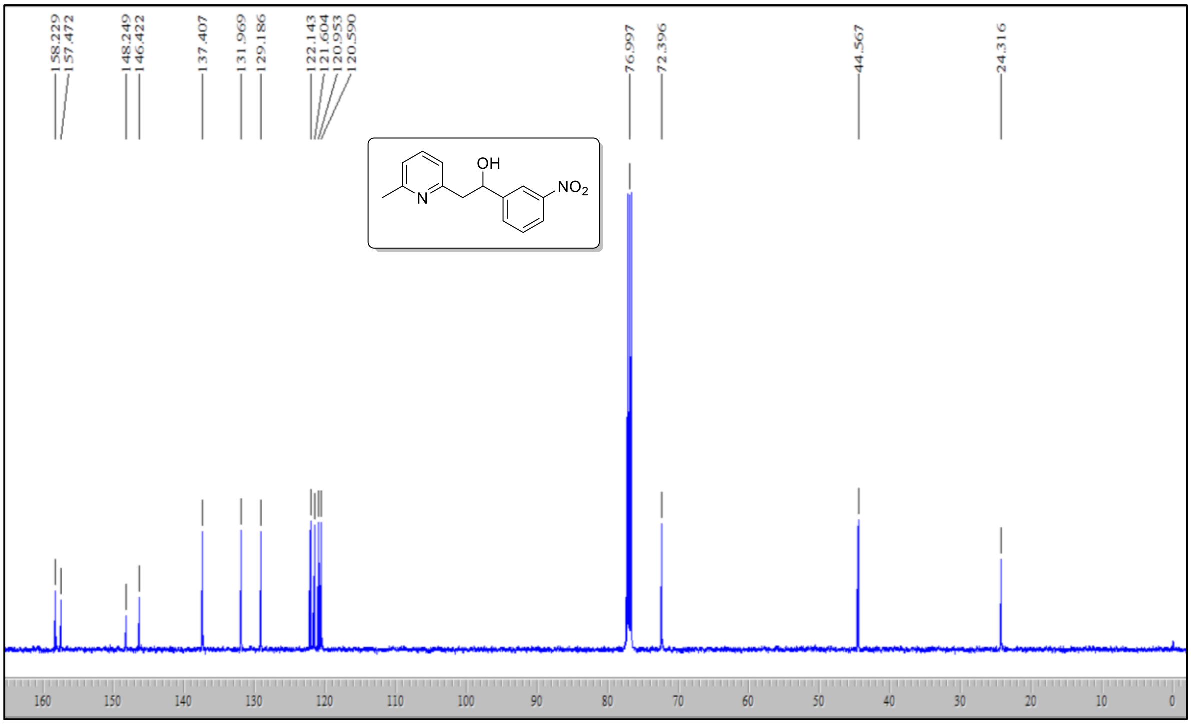

2-(6-Methylpyridin-2-yl)-1-(2-nitrophenyl)ethan-1-ol (3c)

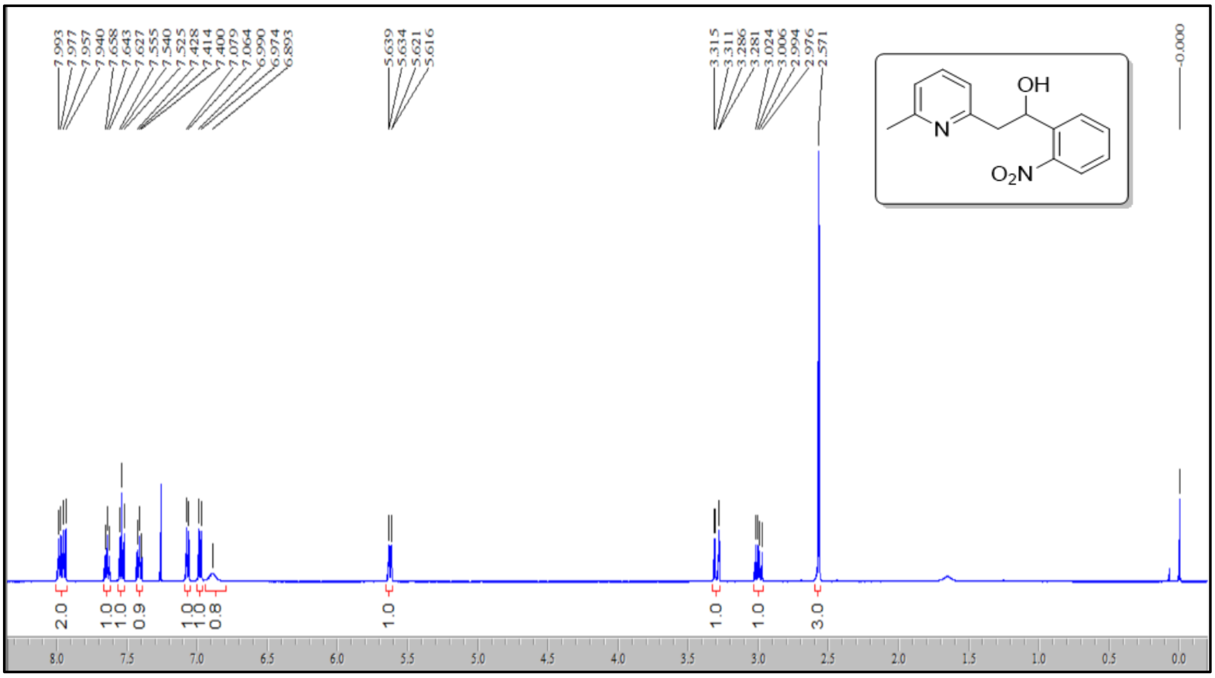

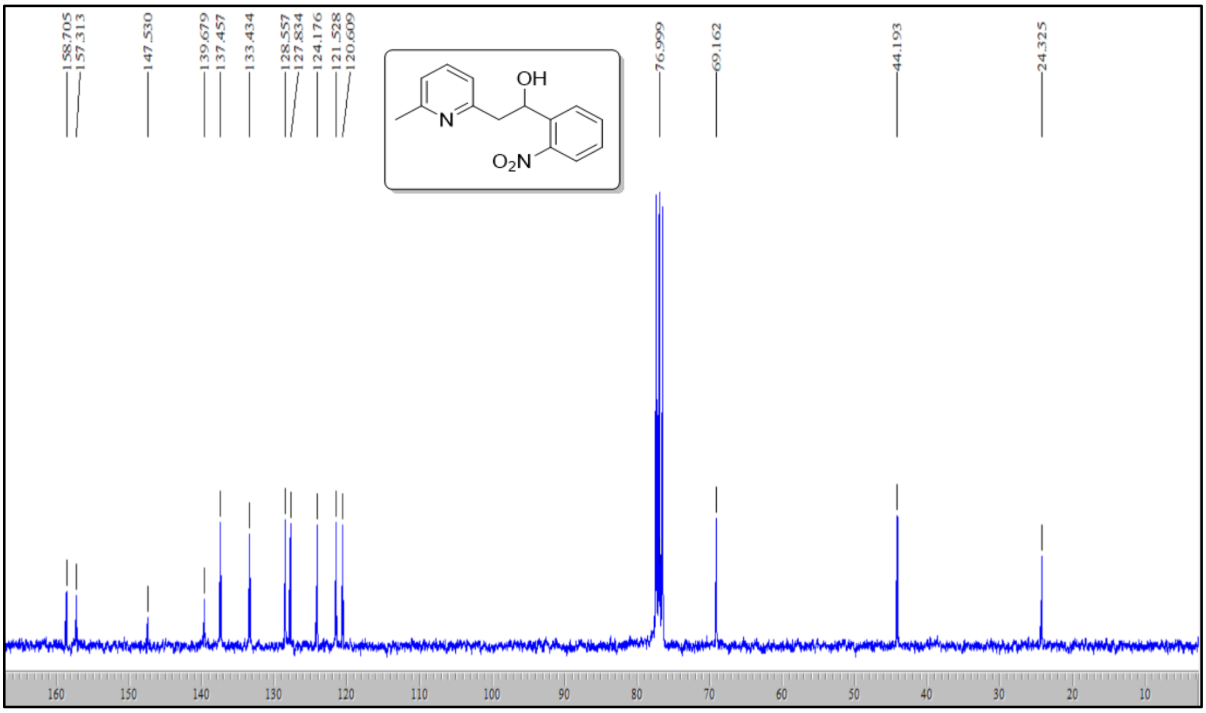

(*E*)-2-Methyl-6-(2-nitrostyryl)pyridine (4c)

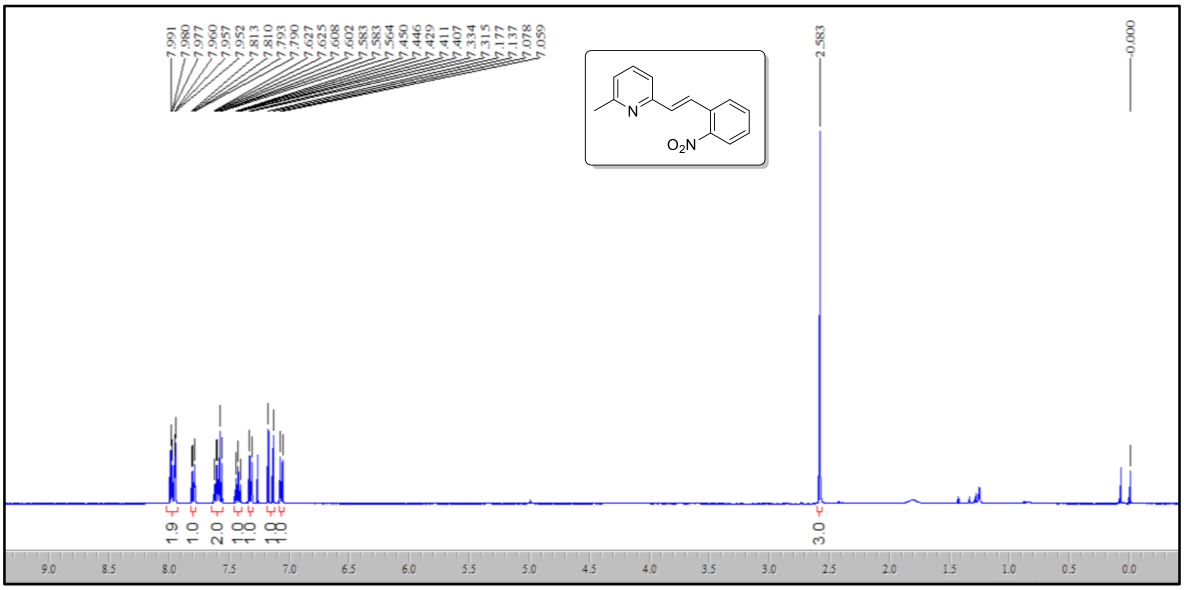

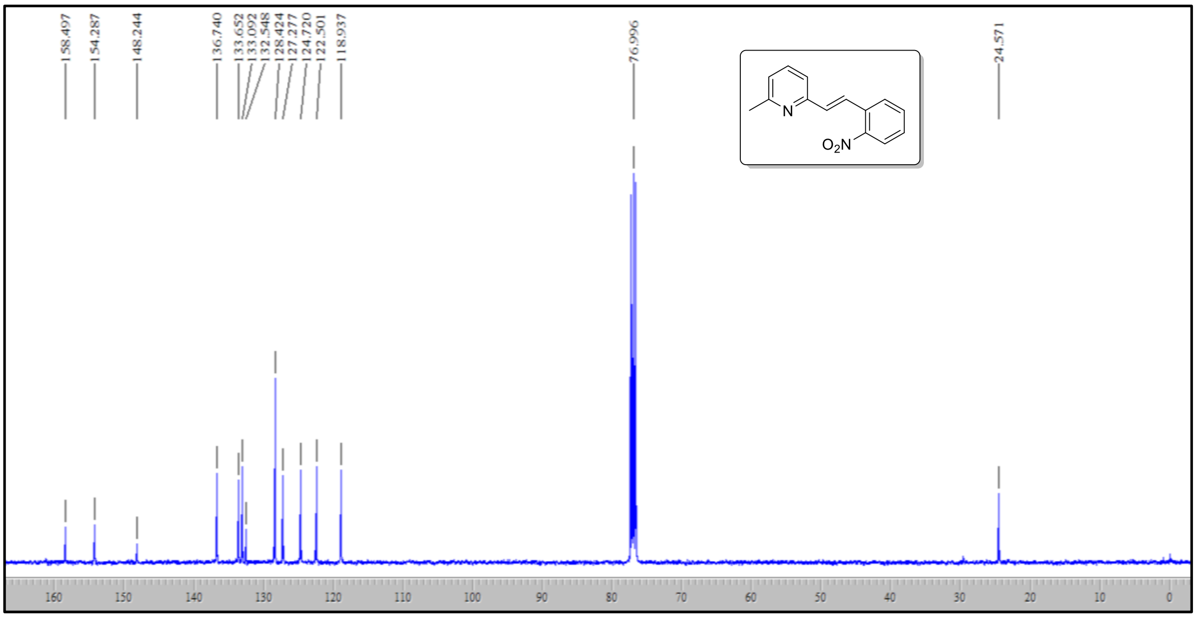

1-(5-Fluoro-2-nitrophenyl)-2-(6-methylpyridin-2-yl)ethan-1-ol (3d)

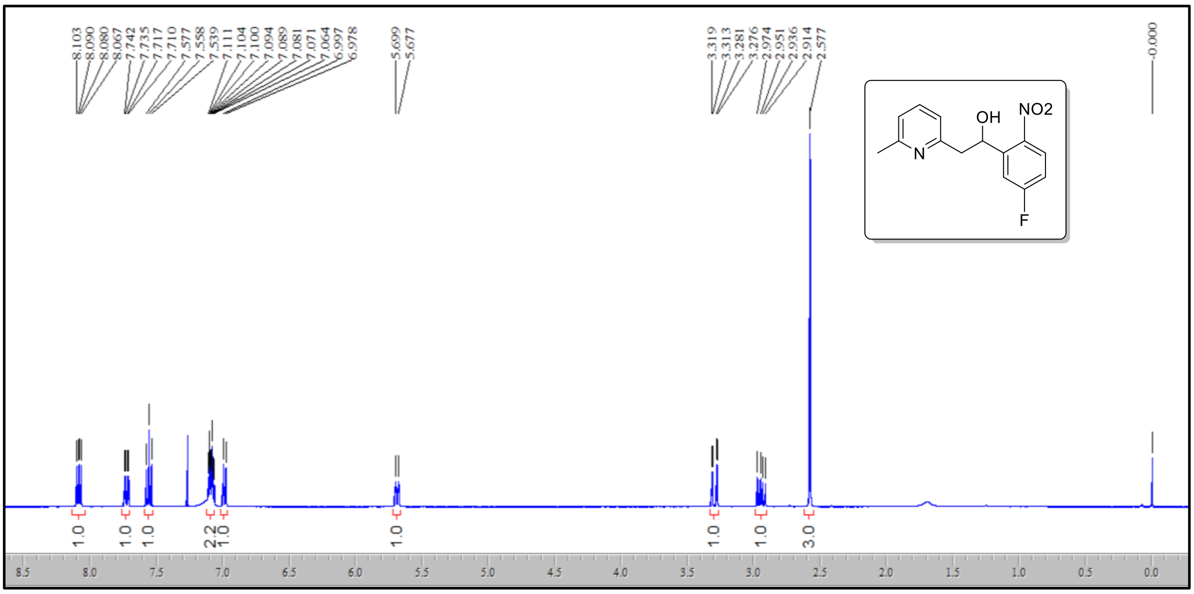

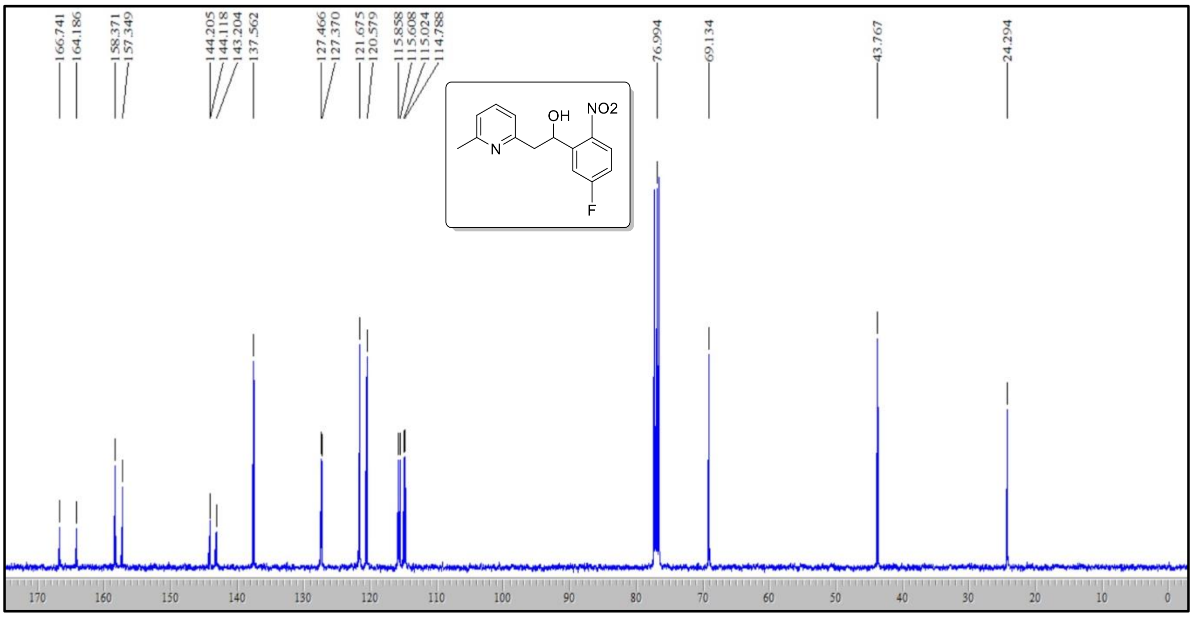

1-(3-Fluorophenyl)-2-(6-methylpyridin-2-yl)ethan-1-ol (3e)

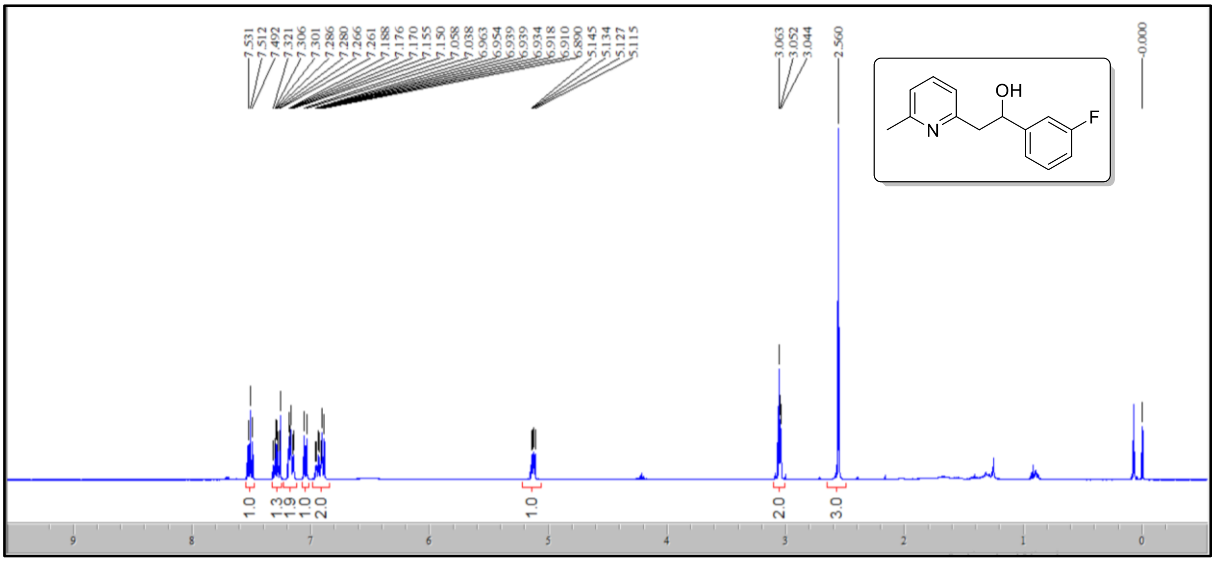

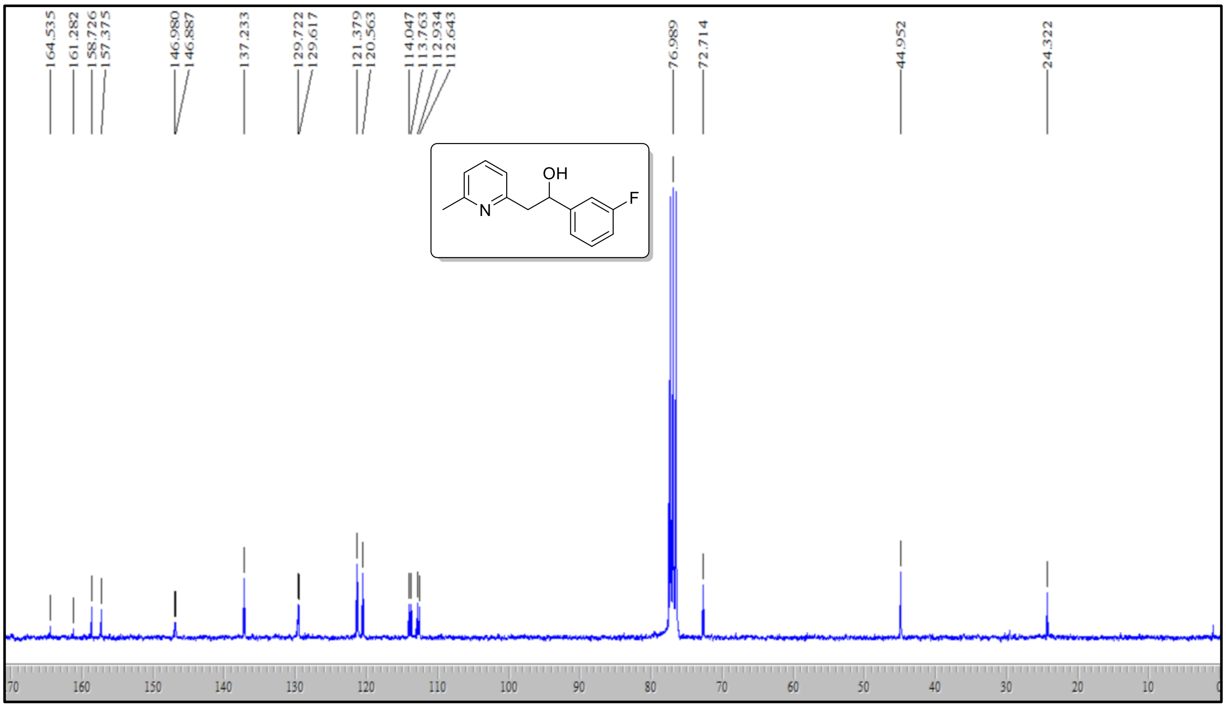

(*E*)-2-(3-Fluorostyryl)-6-methylpyridine (4e)

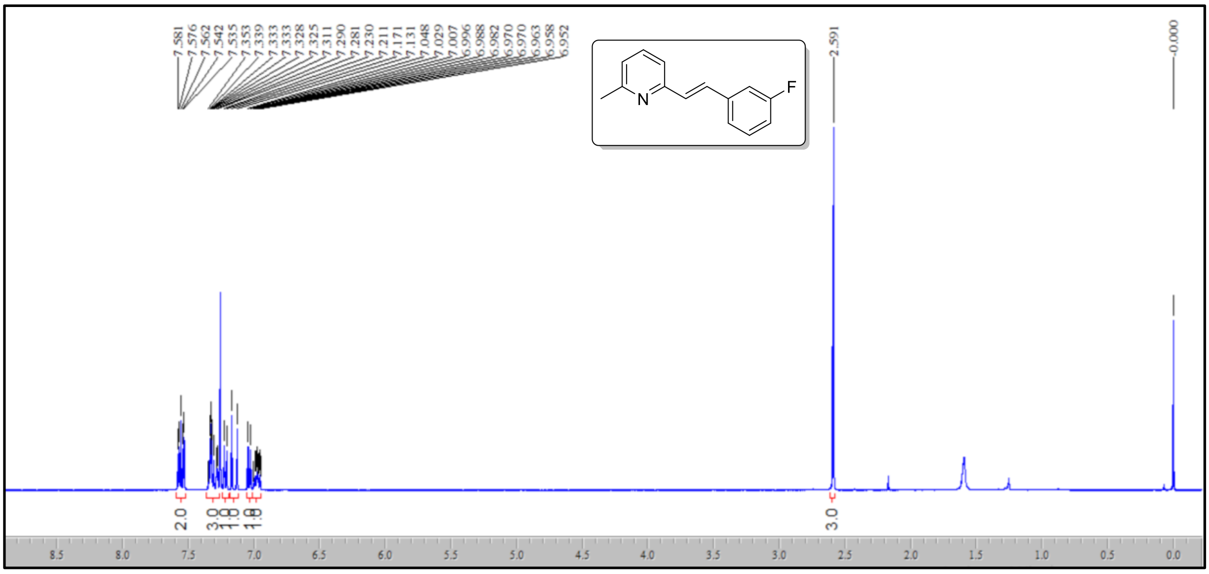

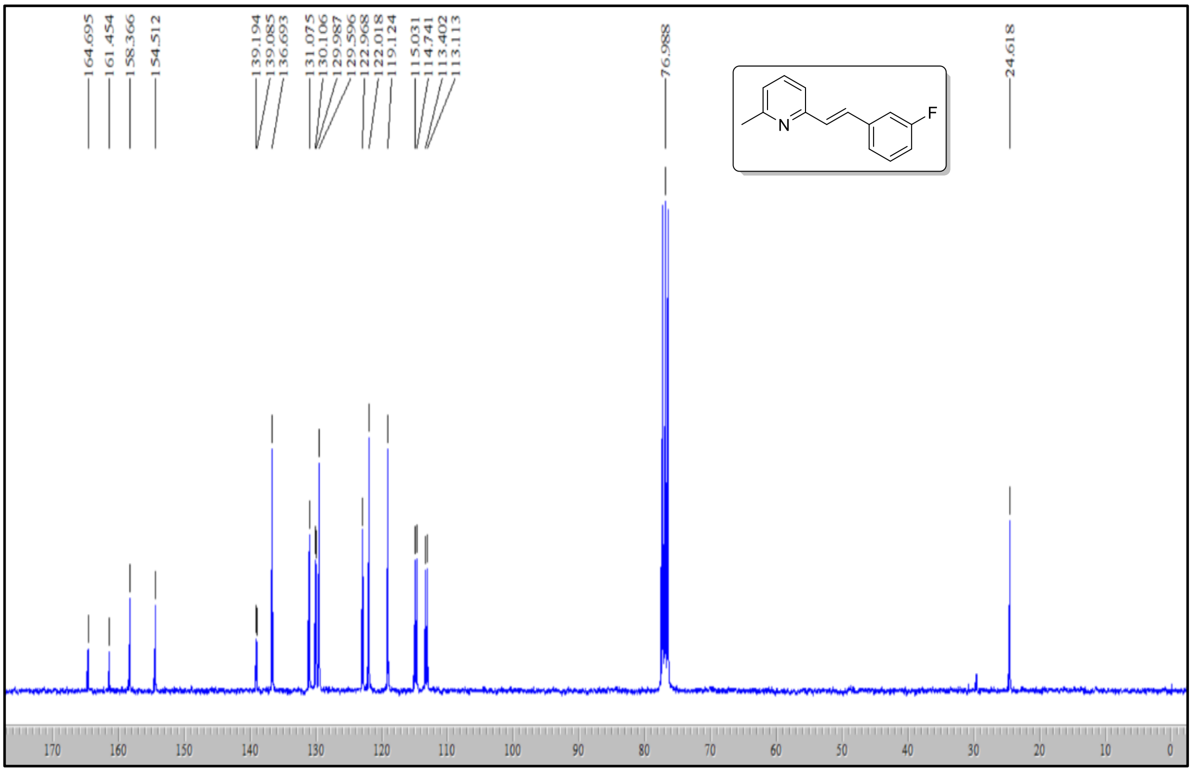

1-(4-Fluorophenyl)-2-(6-methylpyridin-2-yl)ethan-1-ol (3f)

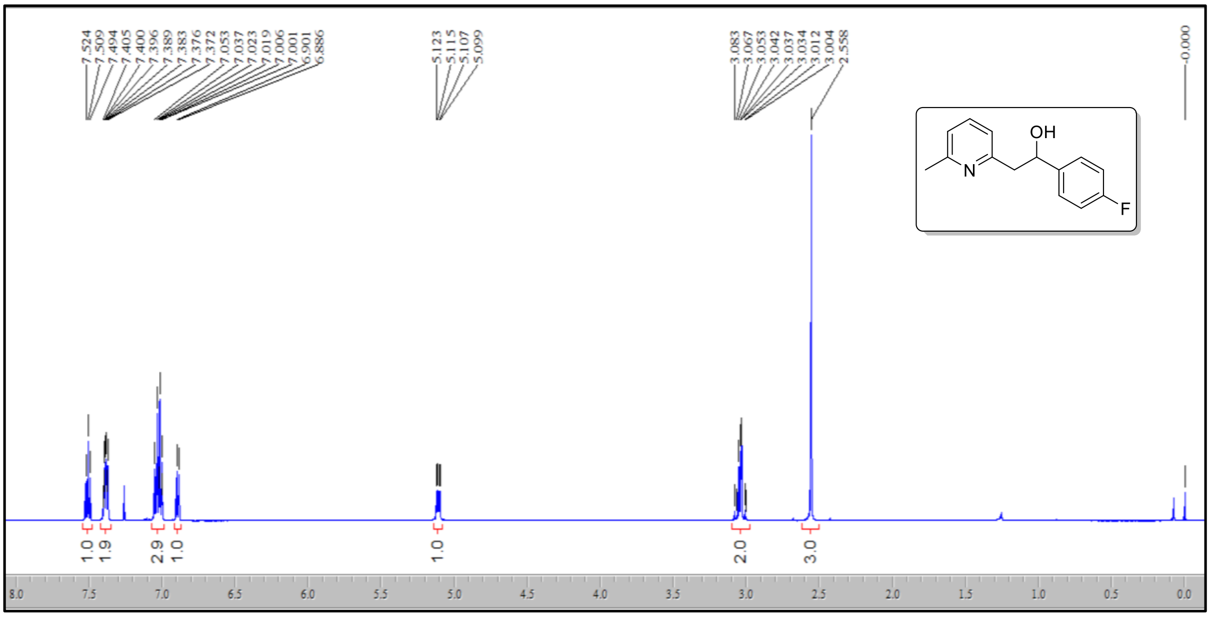

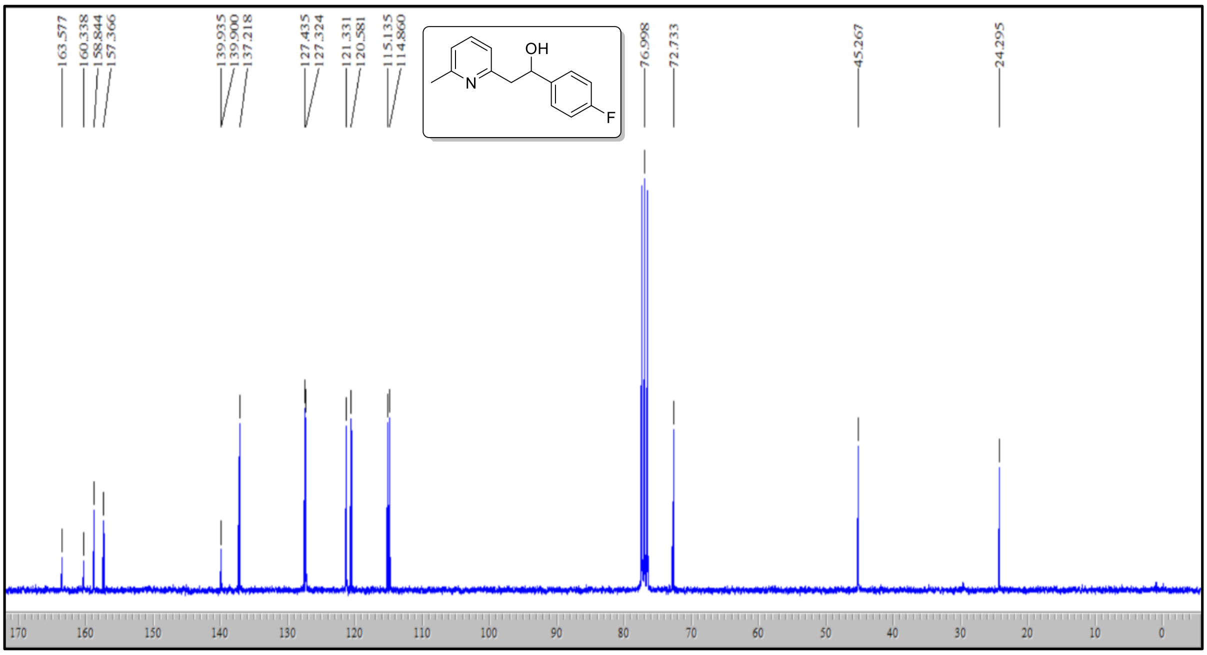

1-(4-Chlorophenyl)-2-(6-methylpyridin-2-yl)ethan-1-ol (3g)

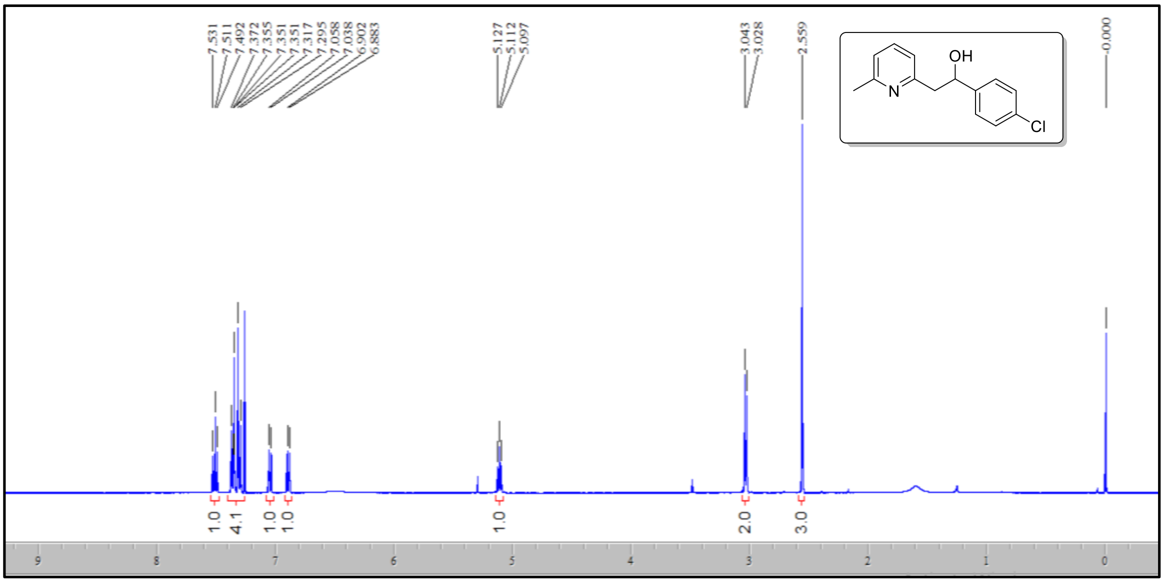

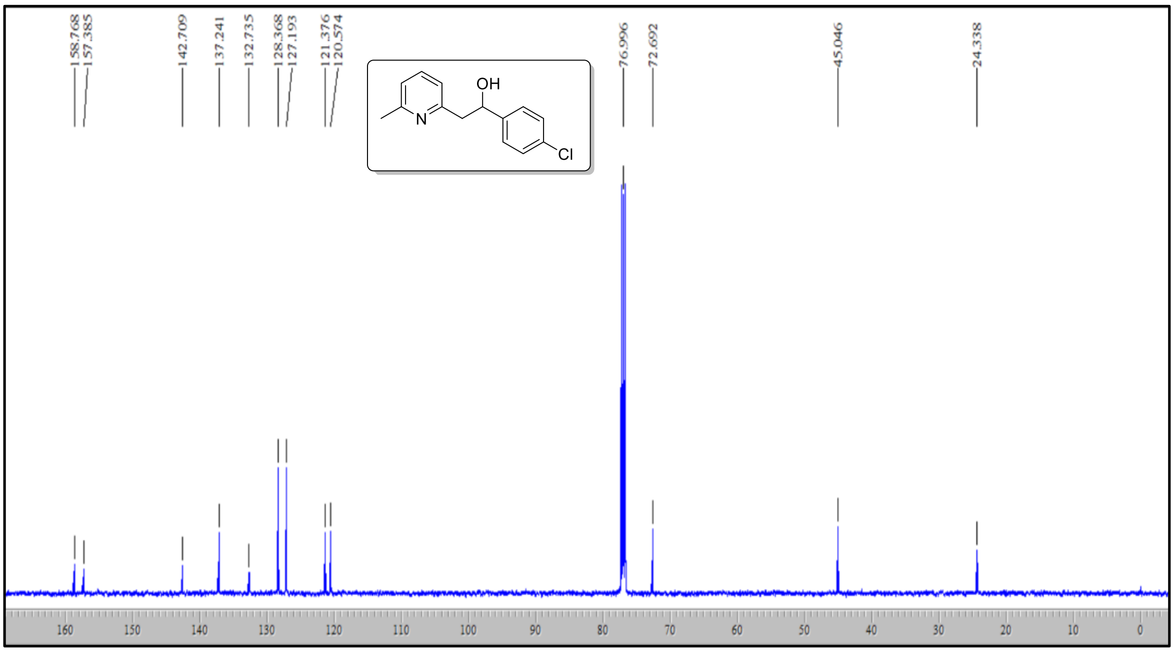

1-(4-Bromophenyl)-2-(6-methylpyridin-2-yl)ethan-1-ol (3h)

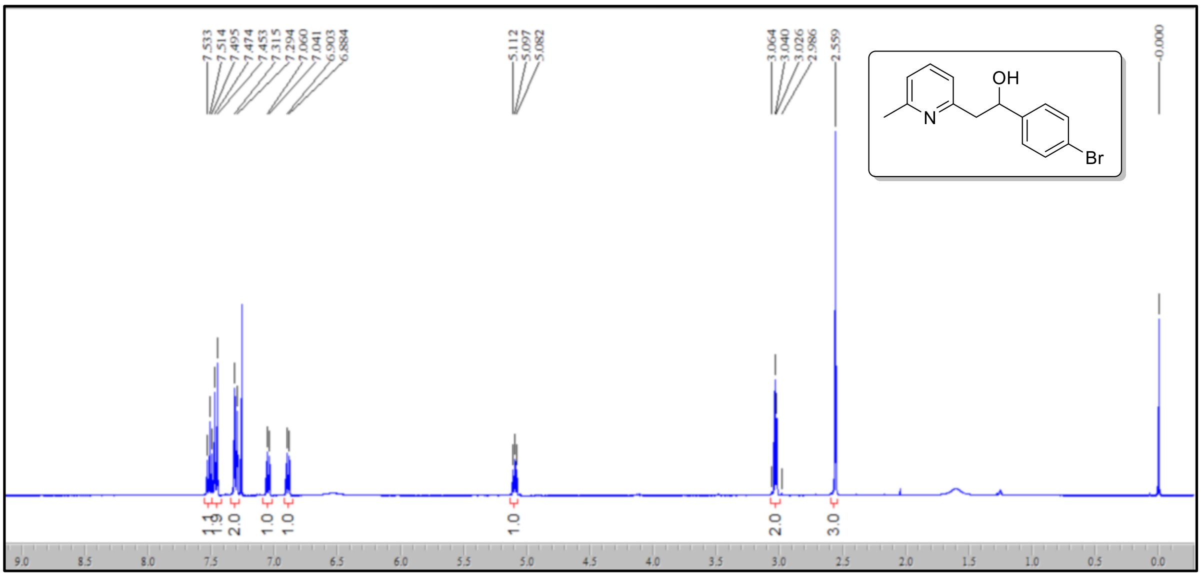

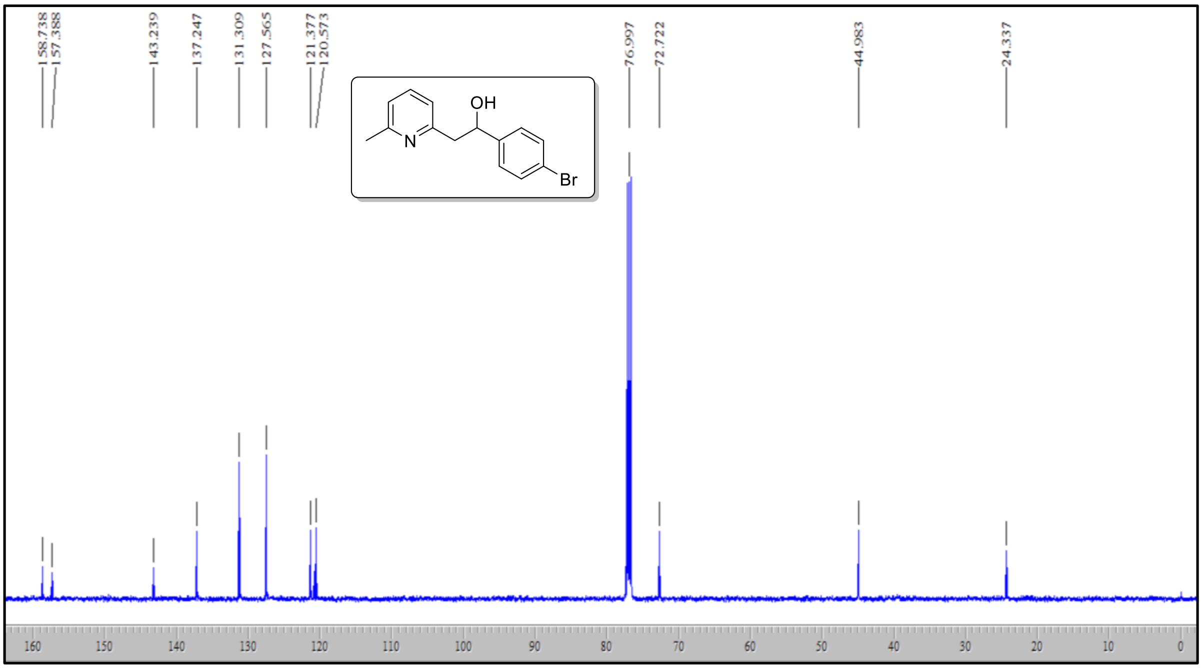

(*E*)-4-(2-(6-Methylpyridin-2-yl)vinyl)benzonitrile (4i)

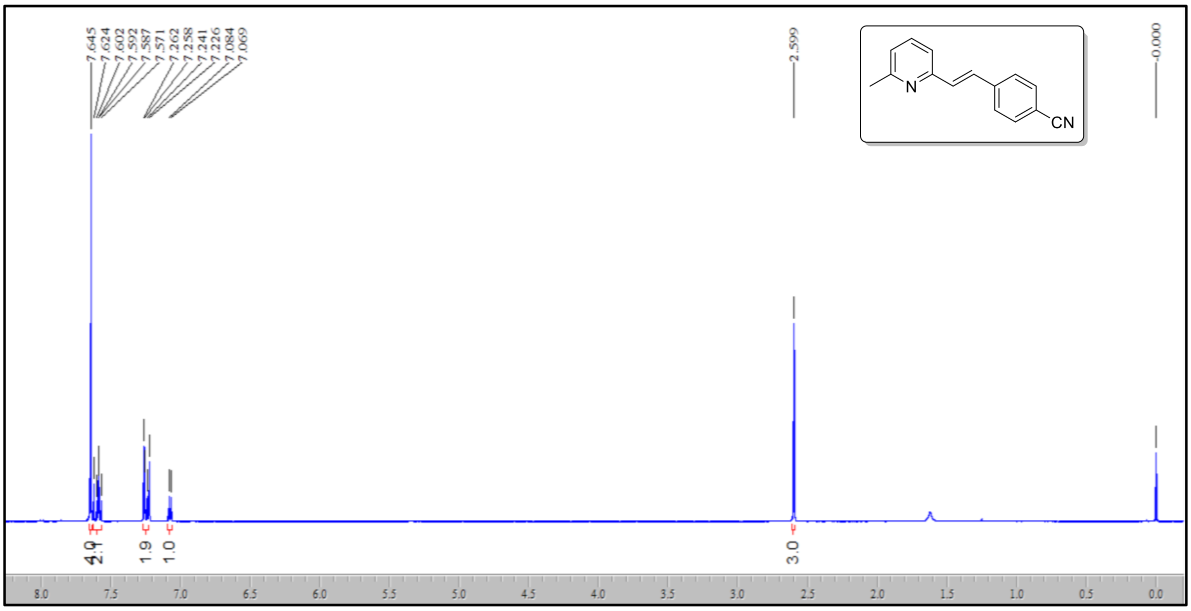

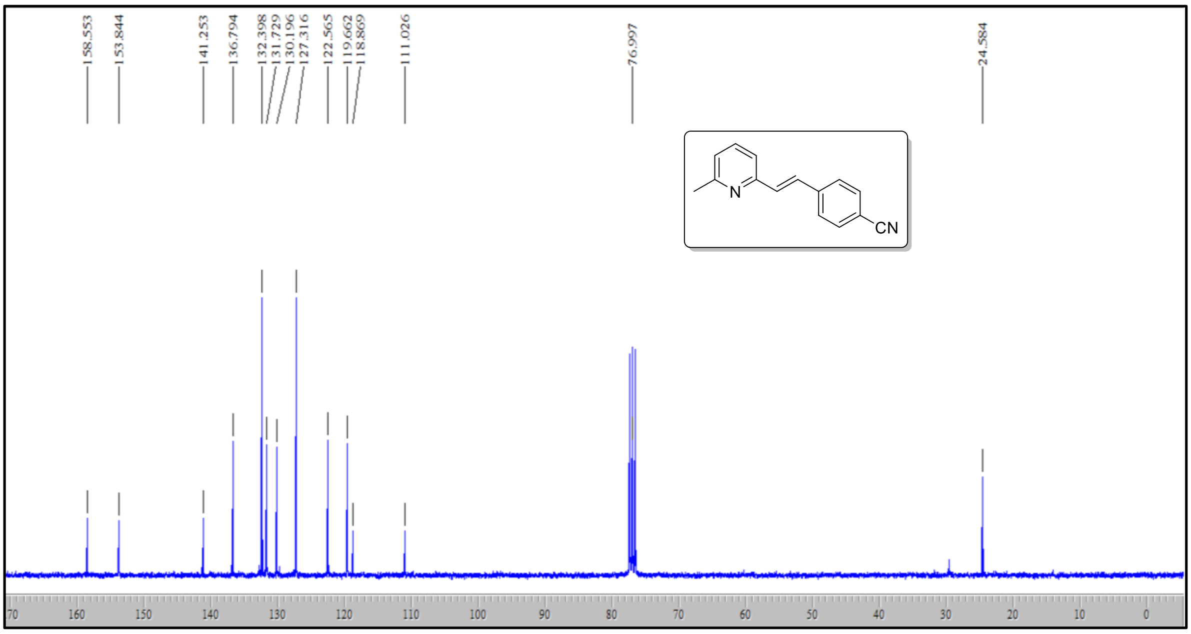

(*E*)-2-Methyl-6-(2-(trifluoromethyl)styryl)pyridine (4j)

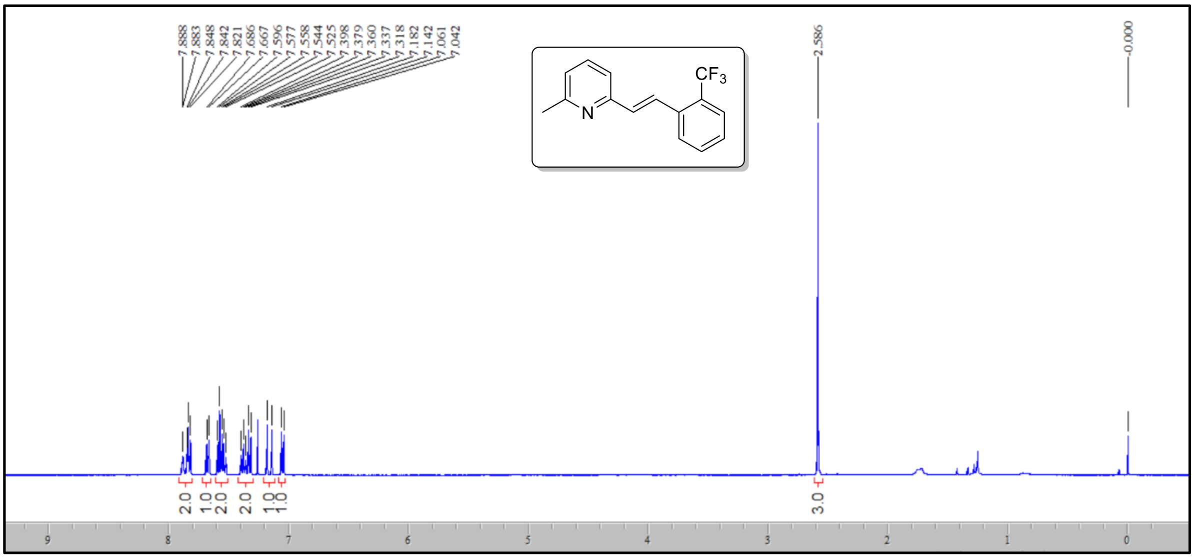

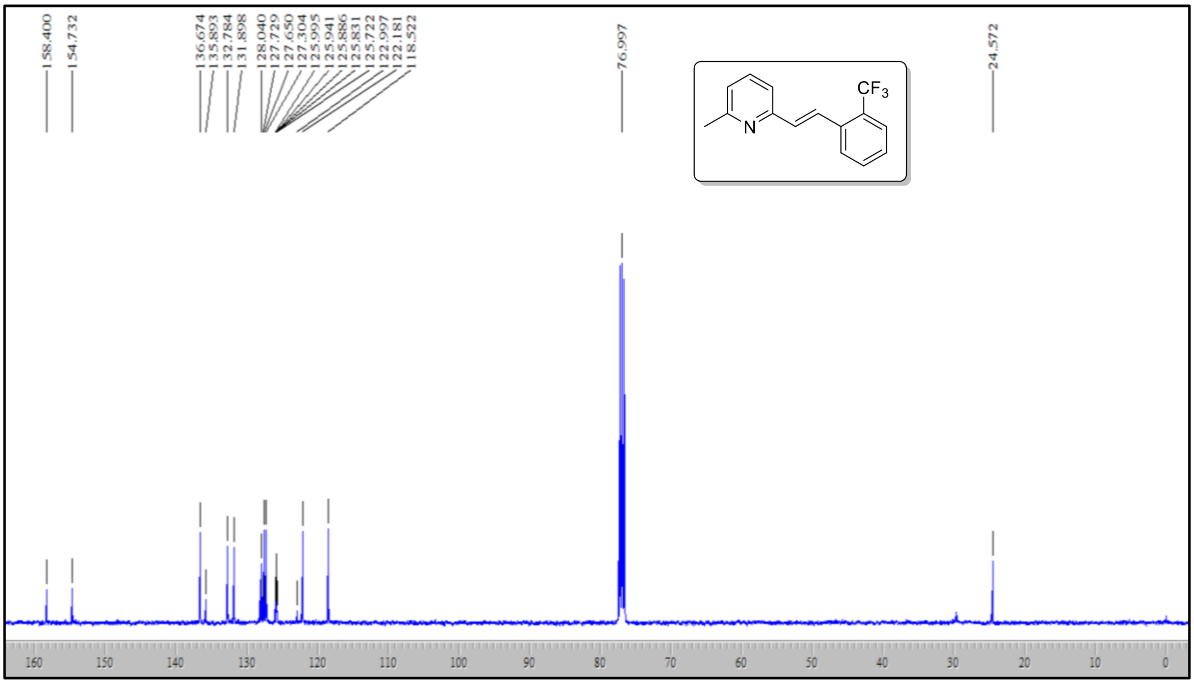

(*E*)-4-(2-(6-Methylpyridin-2-yl)vinyl)benzaldehyde (4k)

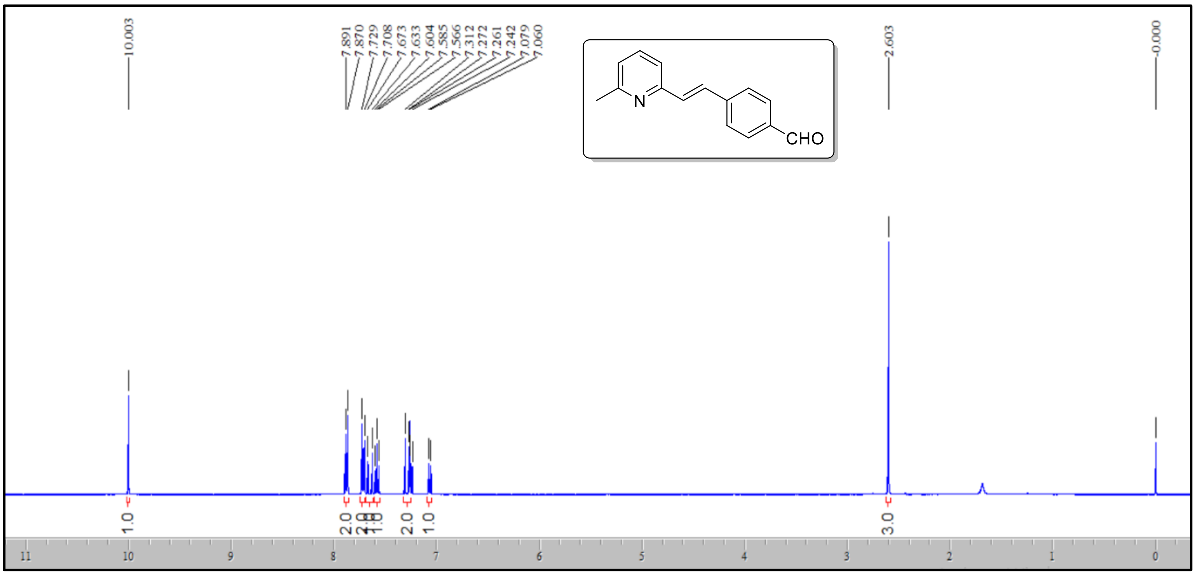

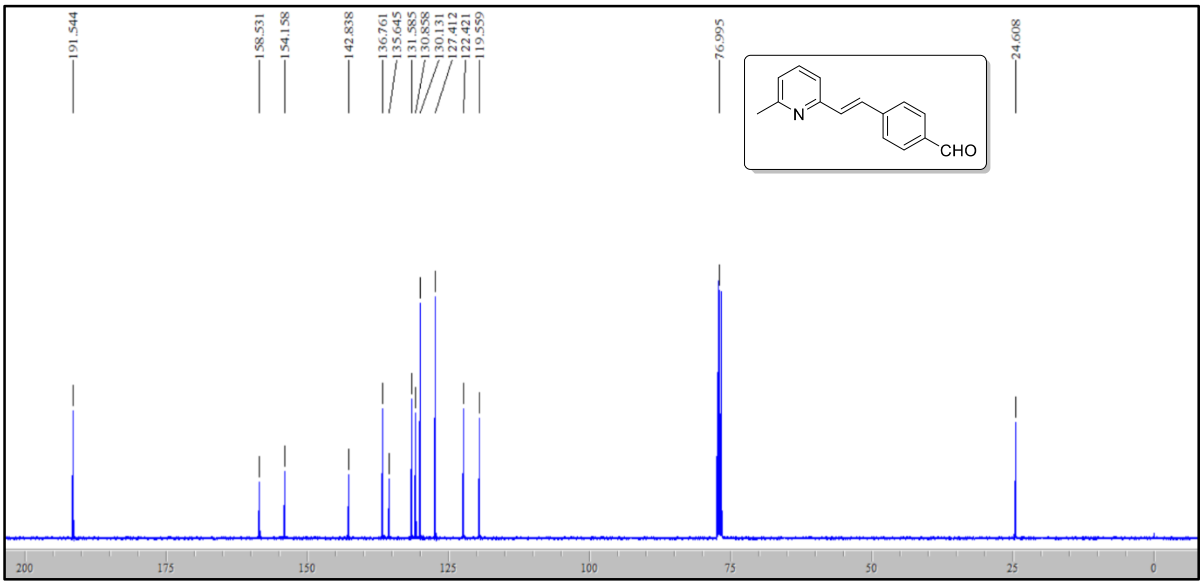

N-(4-(1-Hydroxy-2-(6-methylpyridin-2-yl)ethyl)phenyl)acetamide (3l)

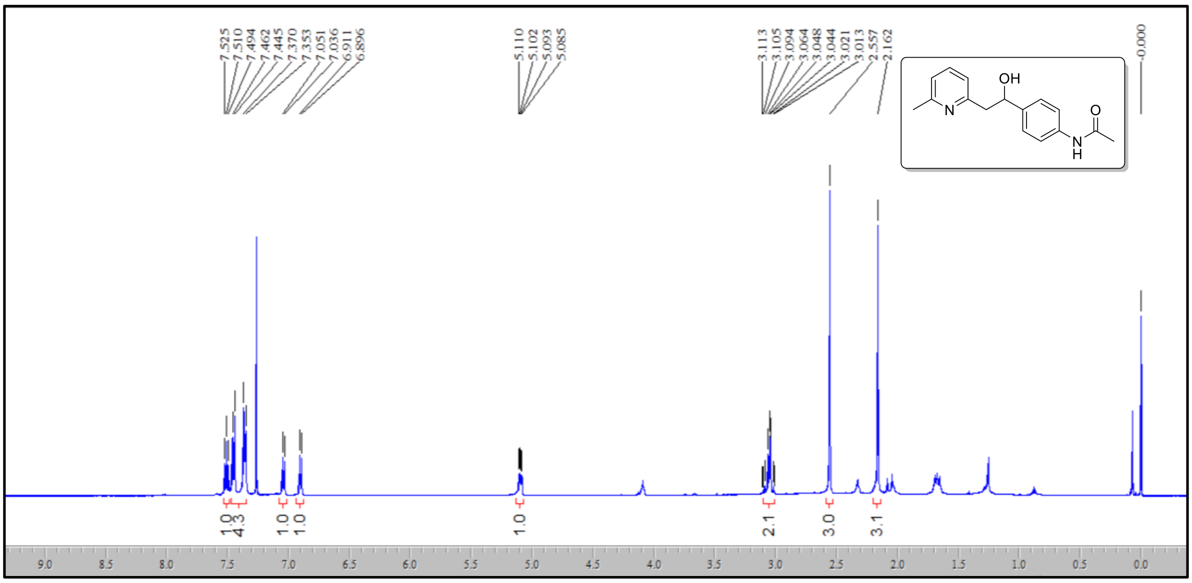

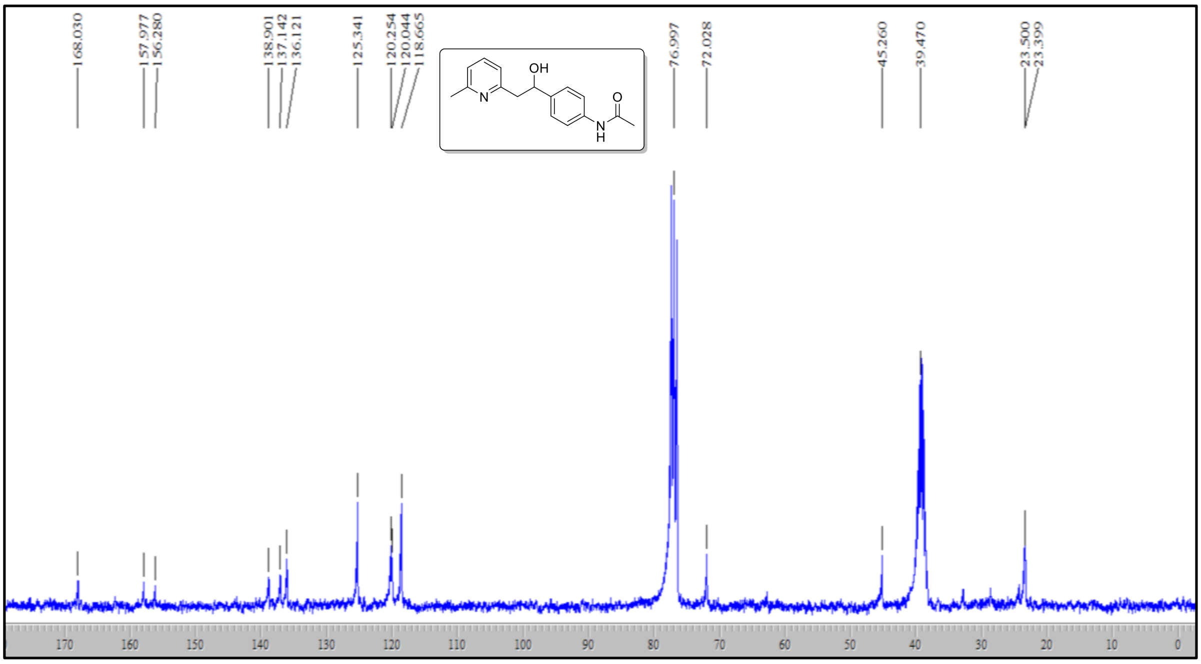

(*E*)-4-(2-(6-Methylpyridin-2-yl)vinyl)phenol (4m)

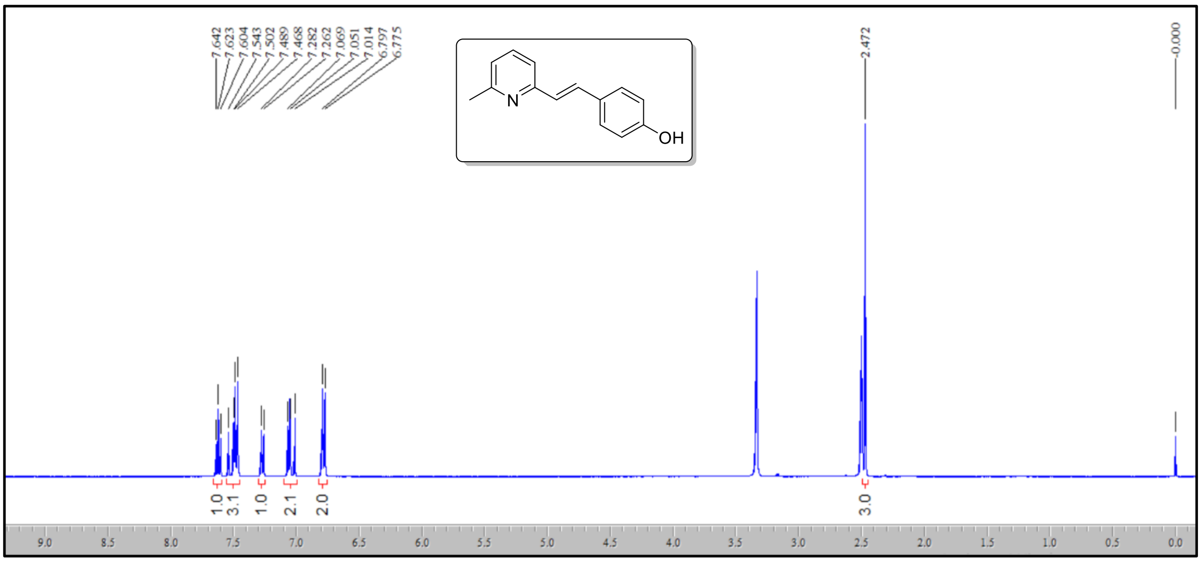

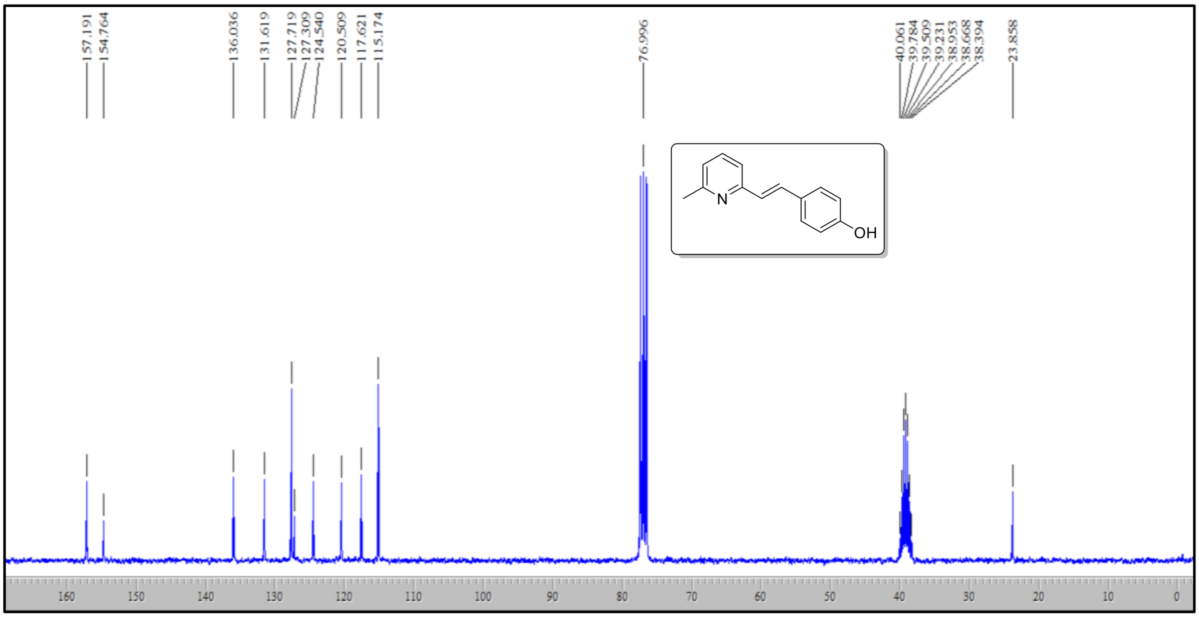

(*E*)-2-(2-(6-Methylpyridin-2-yl)vinyl)phenol (4n)

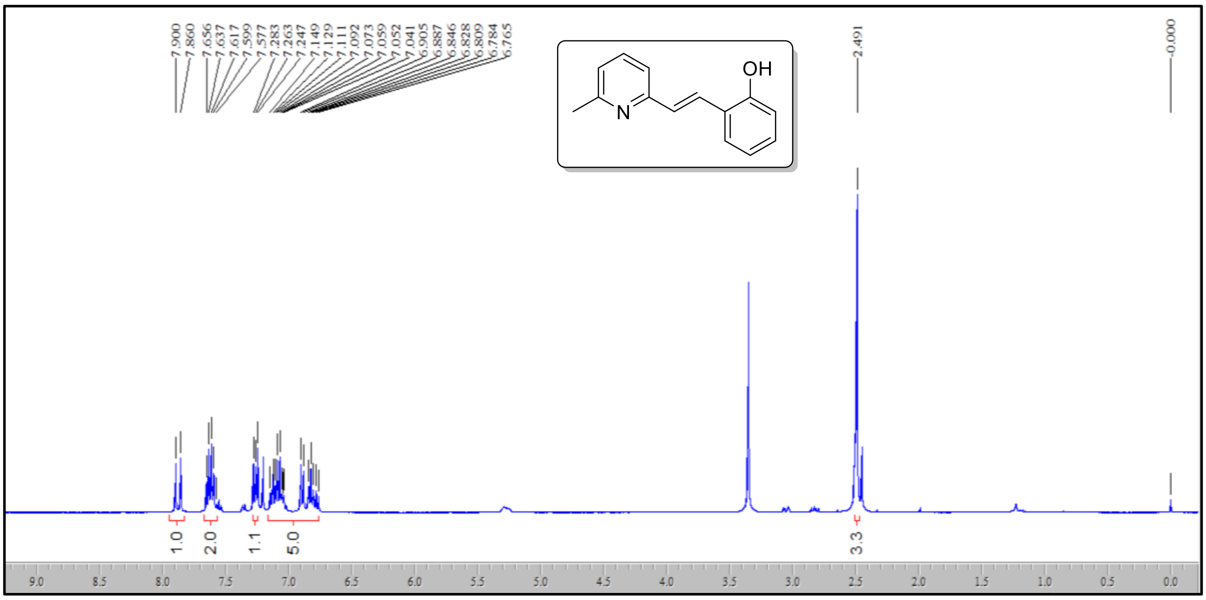

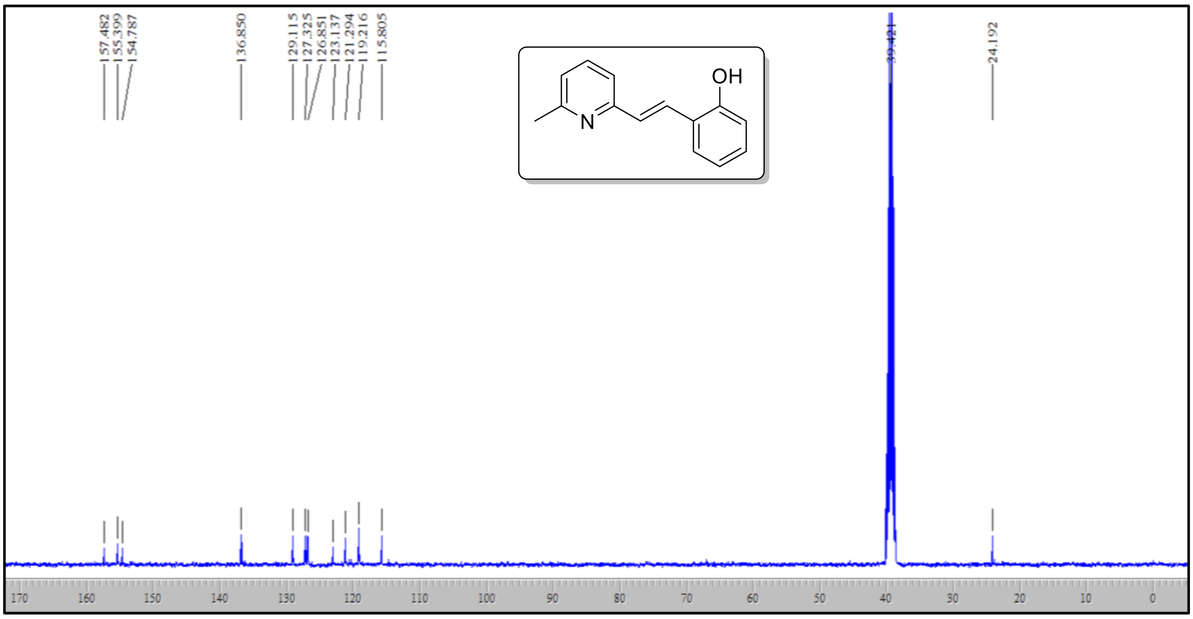

(*E*)-2-Methyl-6-(4-methylstyryl)pyridine (4o)

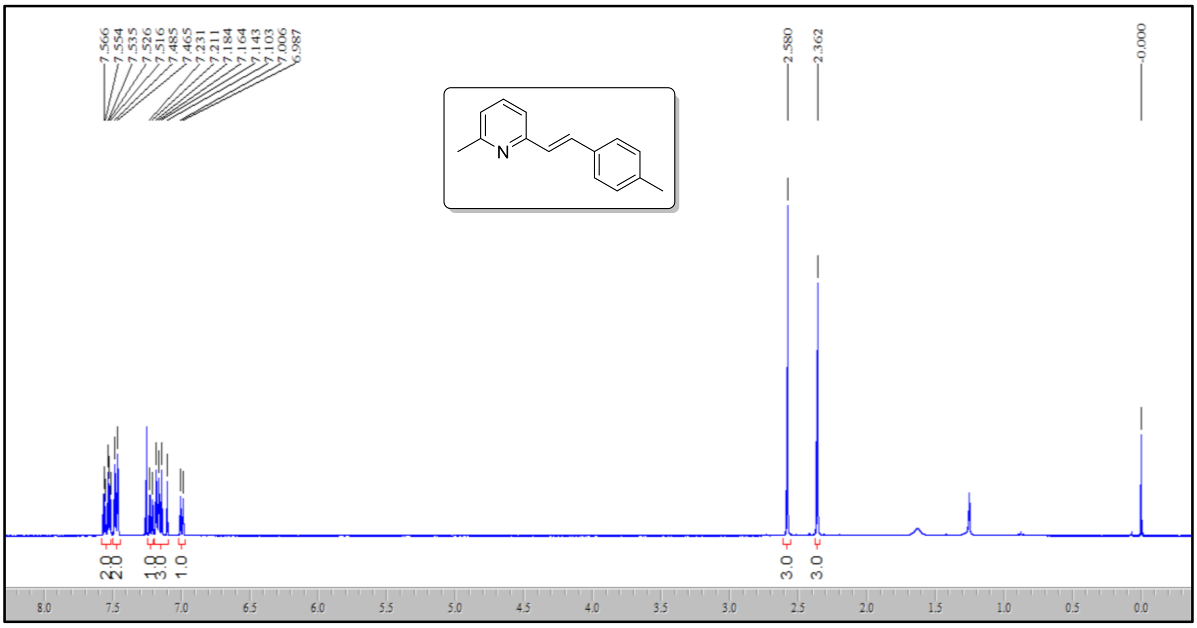

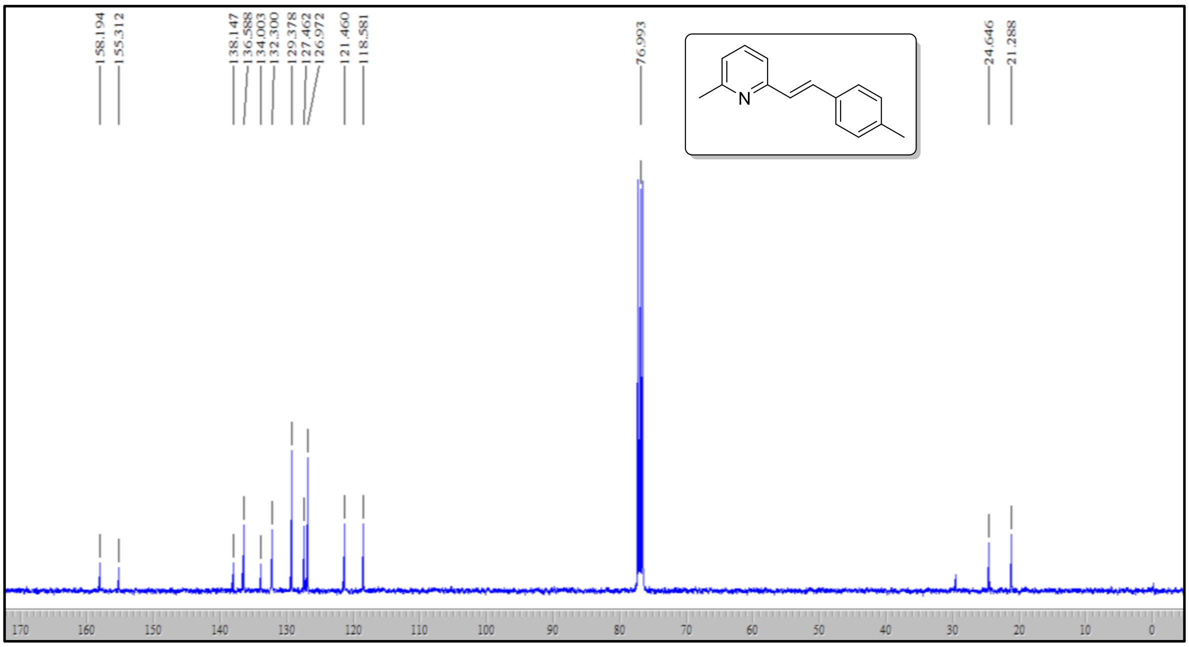

2-(6-Methylpyridin-2-yl)-1-phenylethan-1-ol (3p)

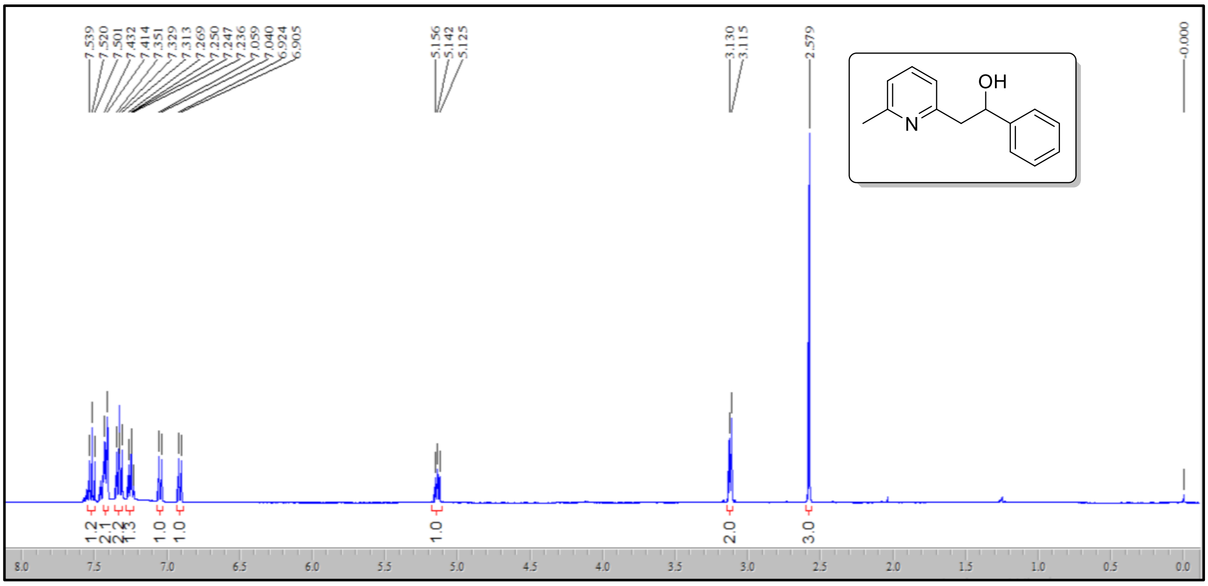

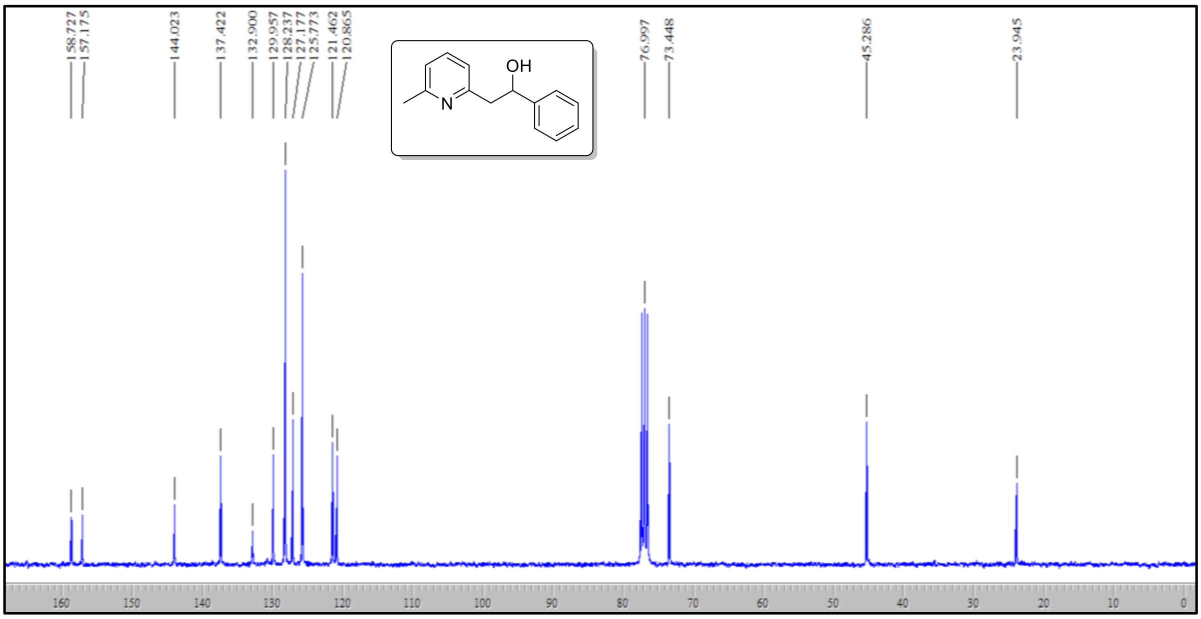

(*E*)-2-Methyl-6-(2-(pyridin-2-yl)vinyl)pyridine (4q)

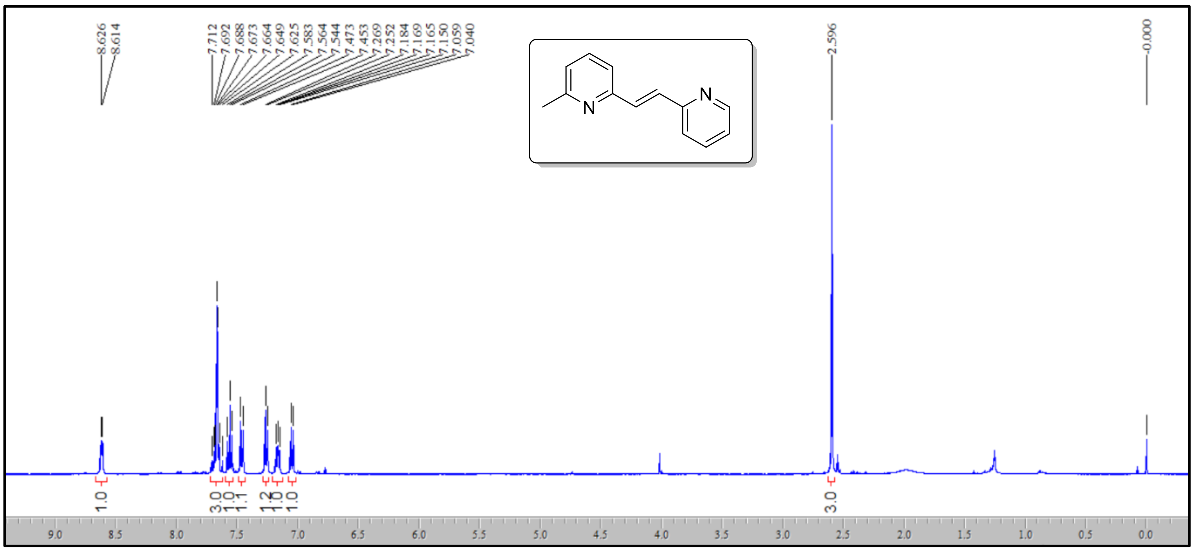

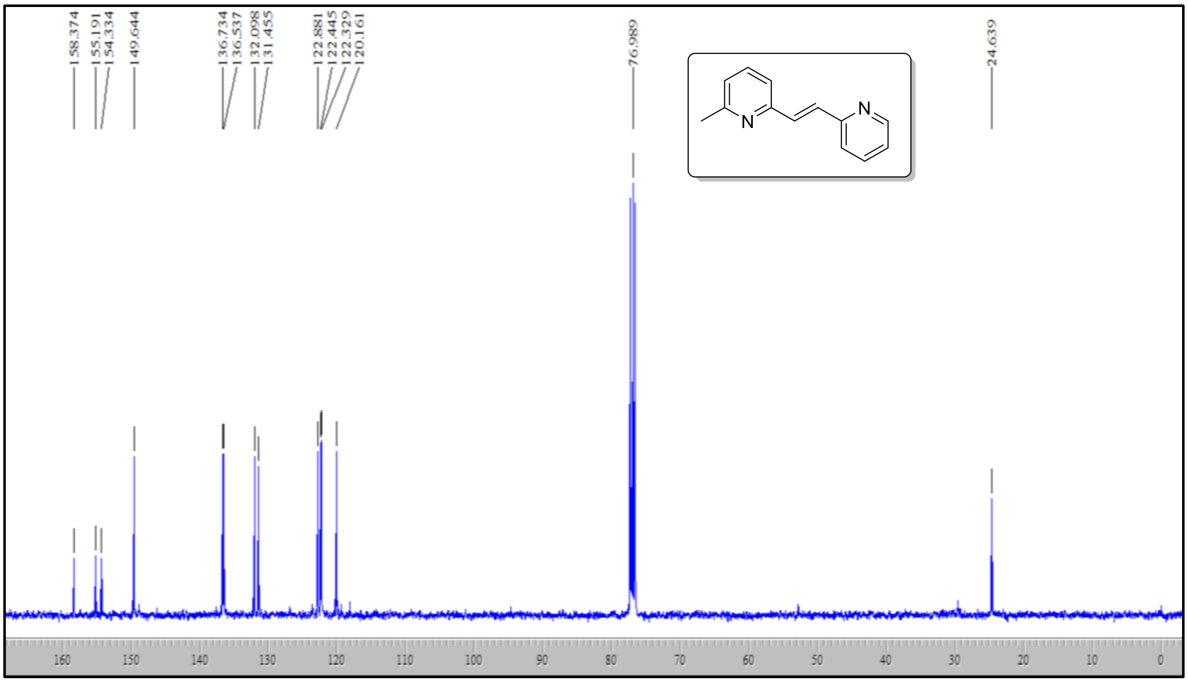

2-(6-Methylpyridin-2-yl)-1-(thiophen-2-yl)ethan-1-ol (3r)

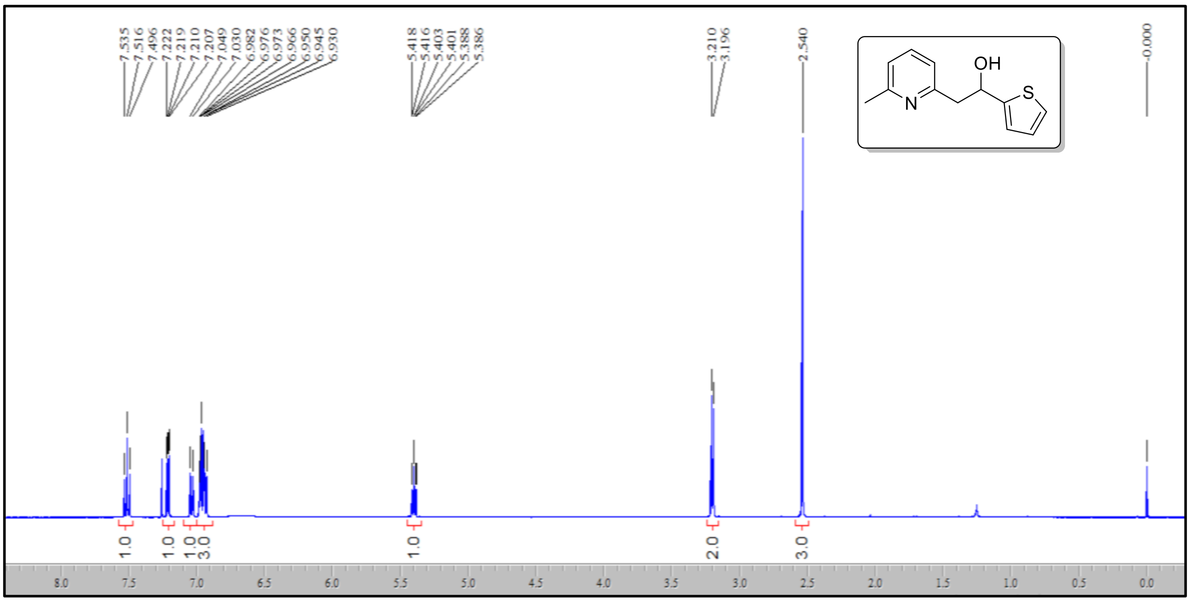

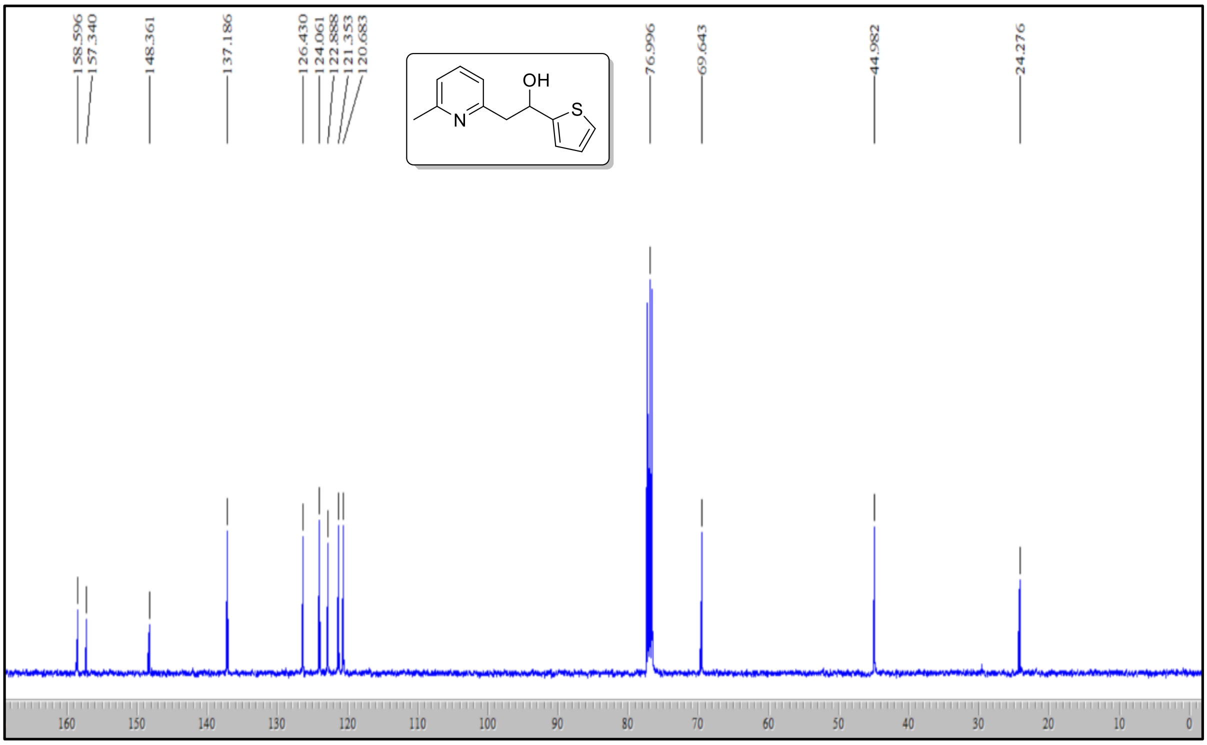

2-(4,6-Dimethylpyridin-2-yl)-1-(4-nitrophenyl)ethan-1-ol (5b)

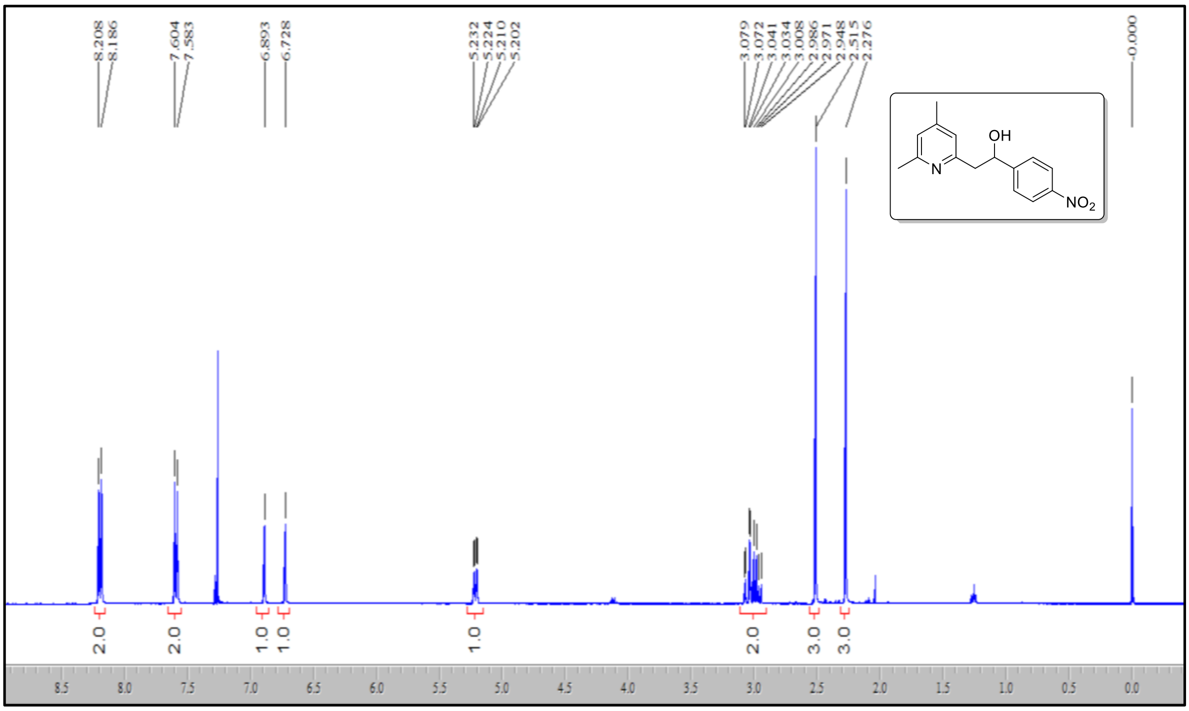

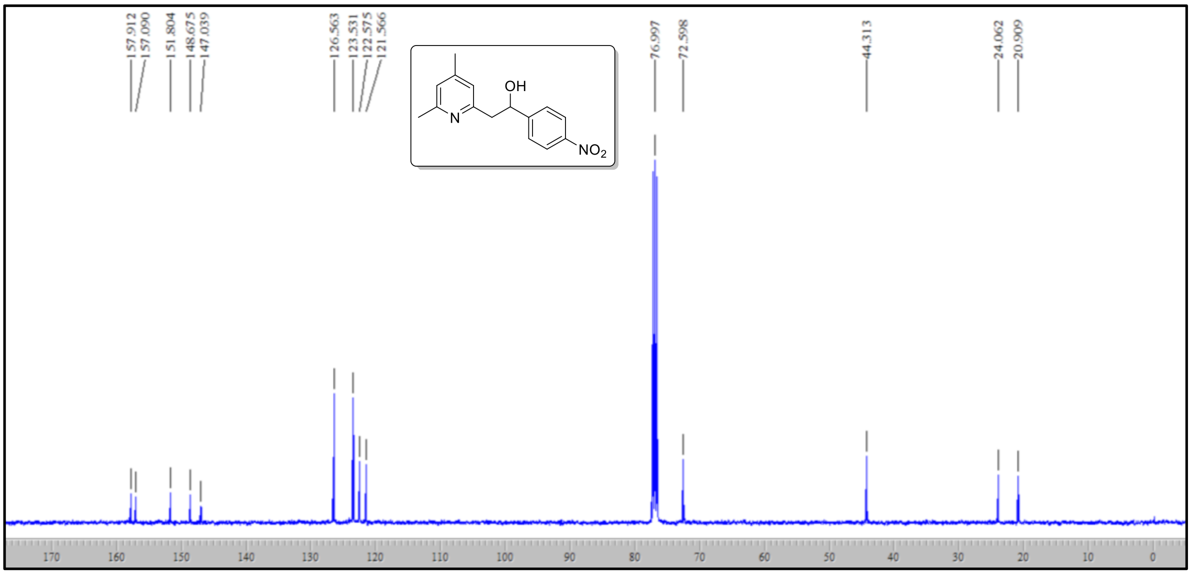

1-(4-Nitrophenyl)-2-(pyridin-2-yl)propan-1-ol (5c)

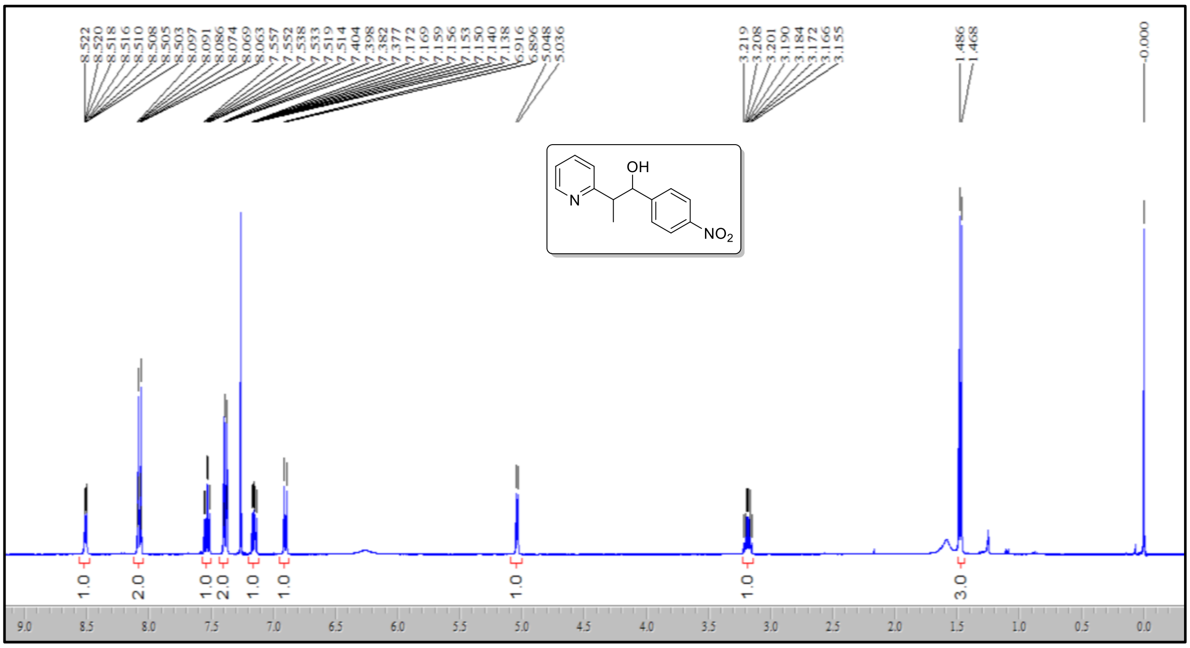

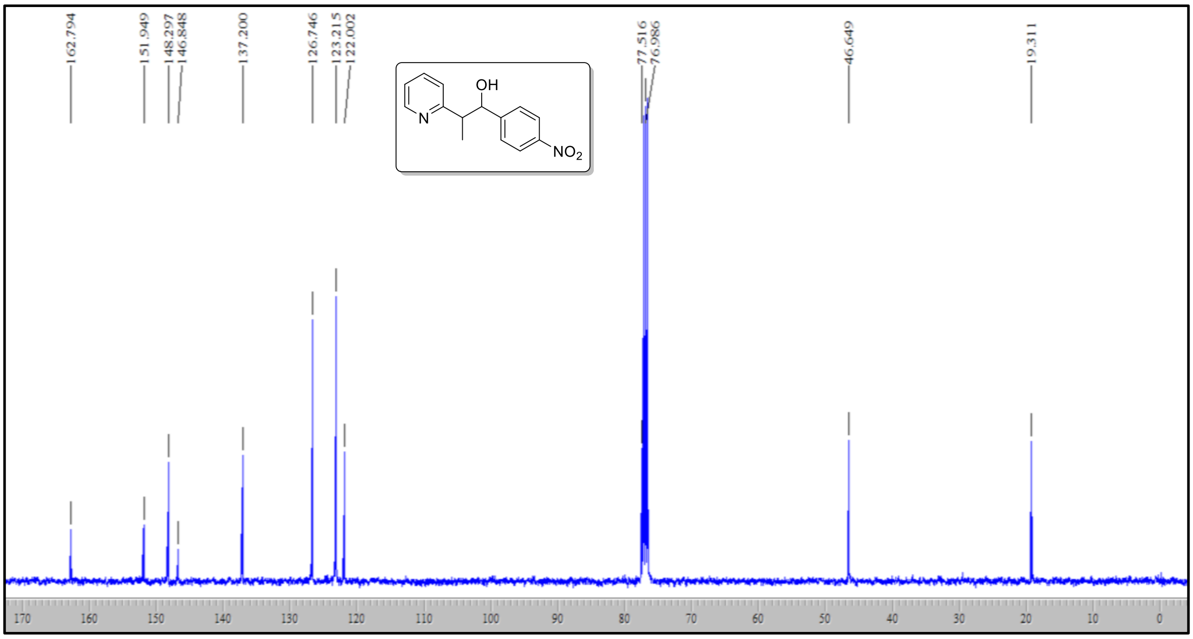

1-(4-Nitrophenyl)-2-(pyridin-2-yl)ethan-1-ol (5d)

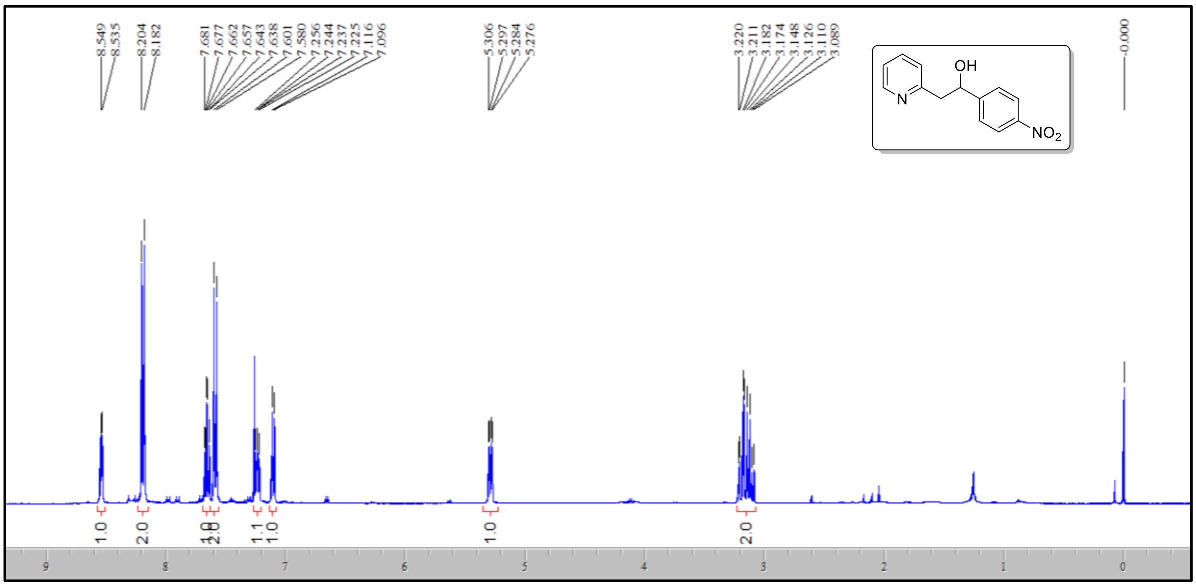

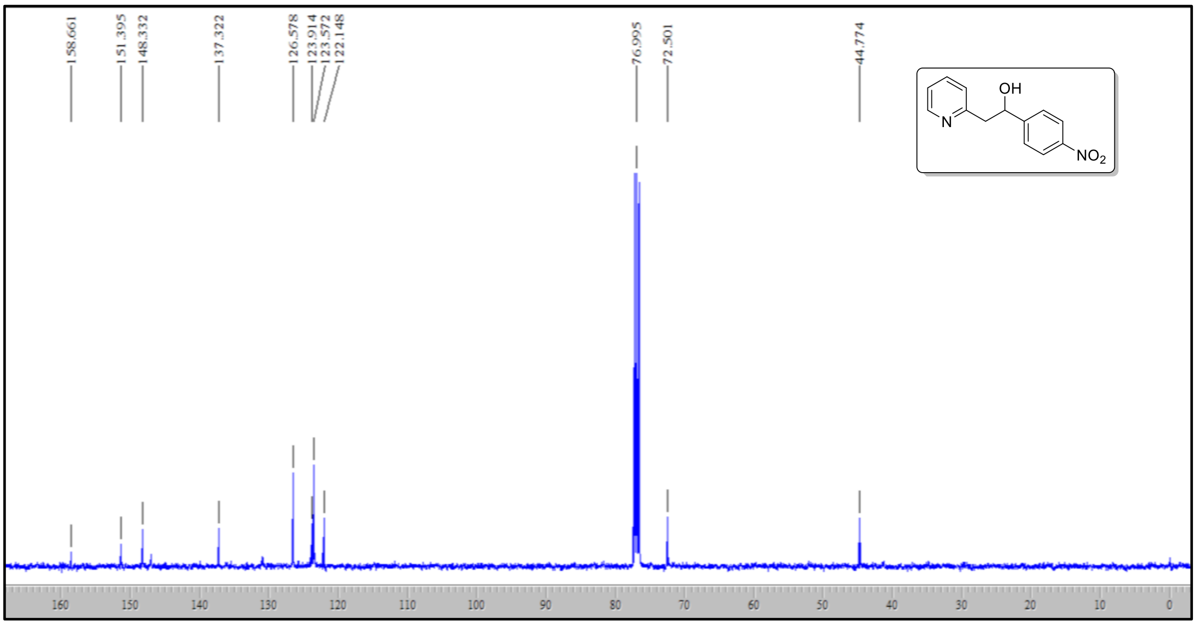

(*E*)-2-(4-Nitrostyryl)pyridine (6d)

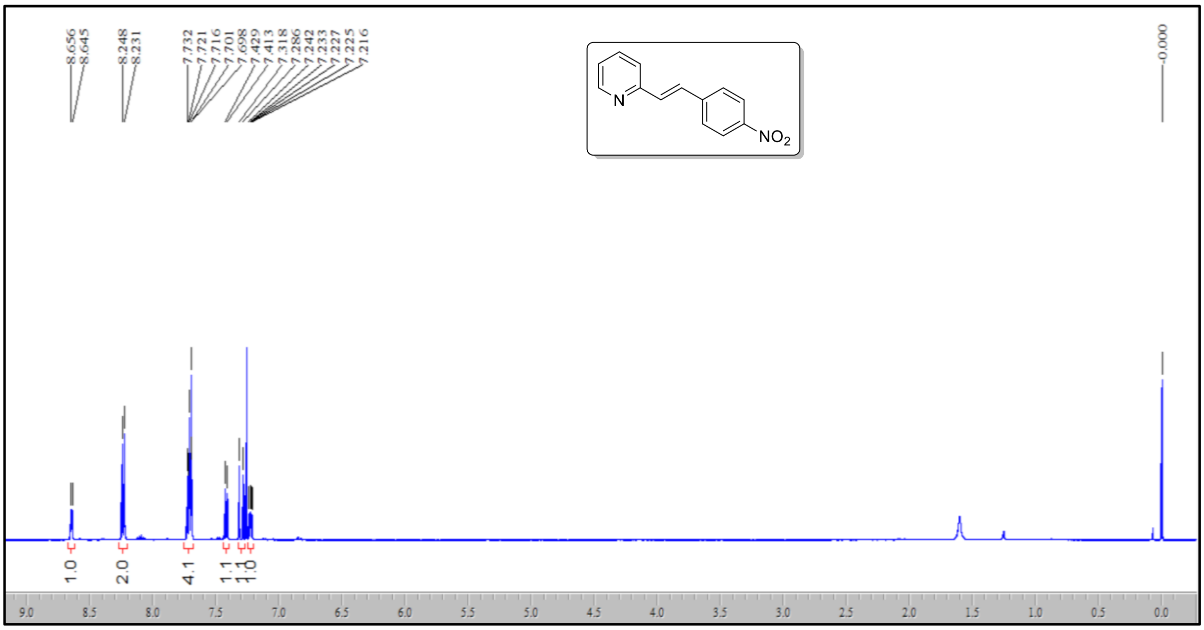

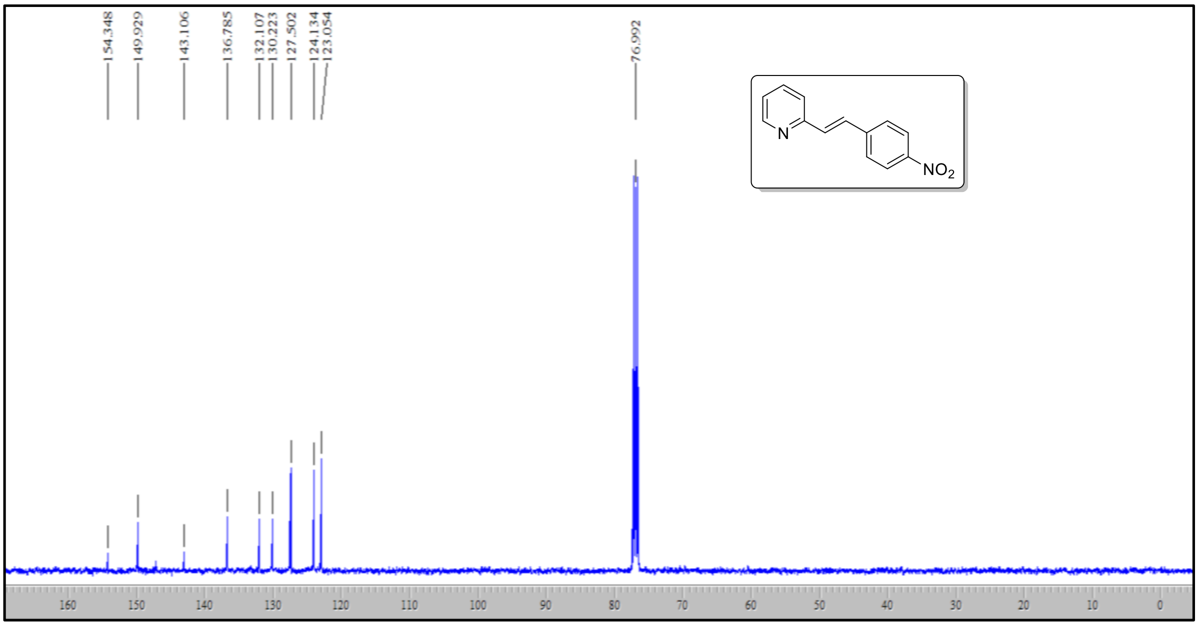

(*E*)-2-(4-Nitrostyryl)quinoline (6f)

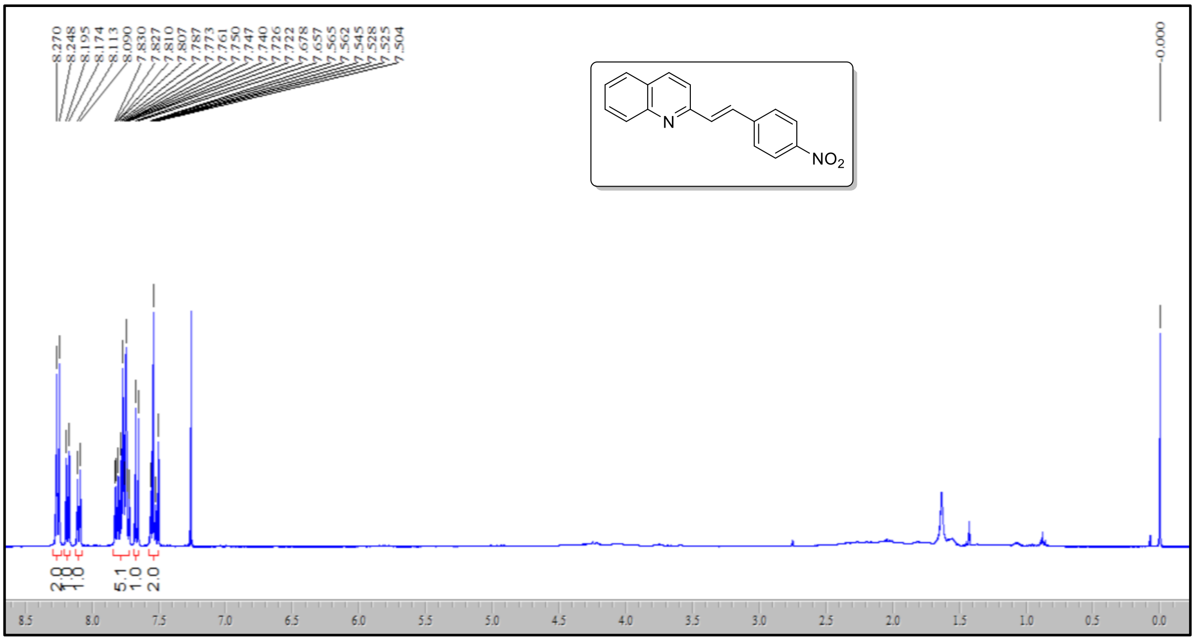

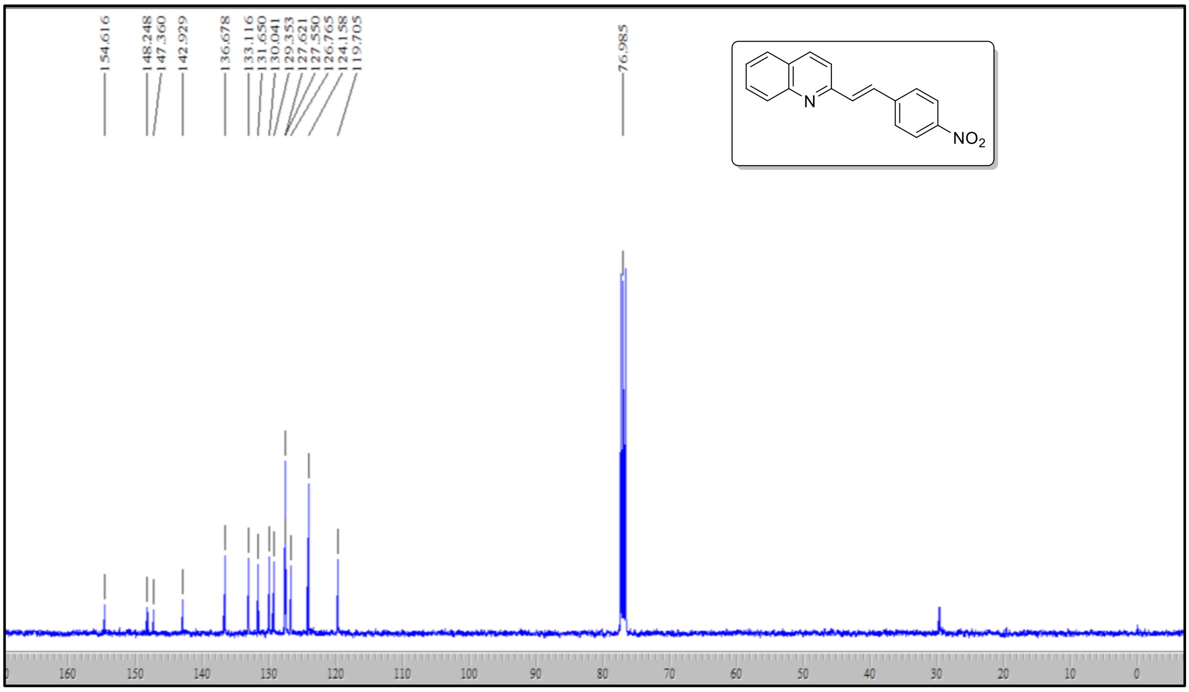

(*E*)-6-Fluoro-2-(4- nitrostyryl)quinoline (6g)

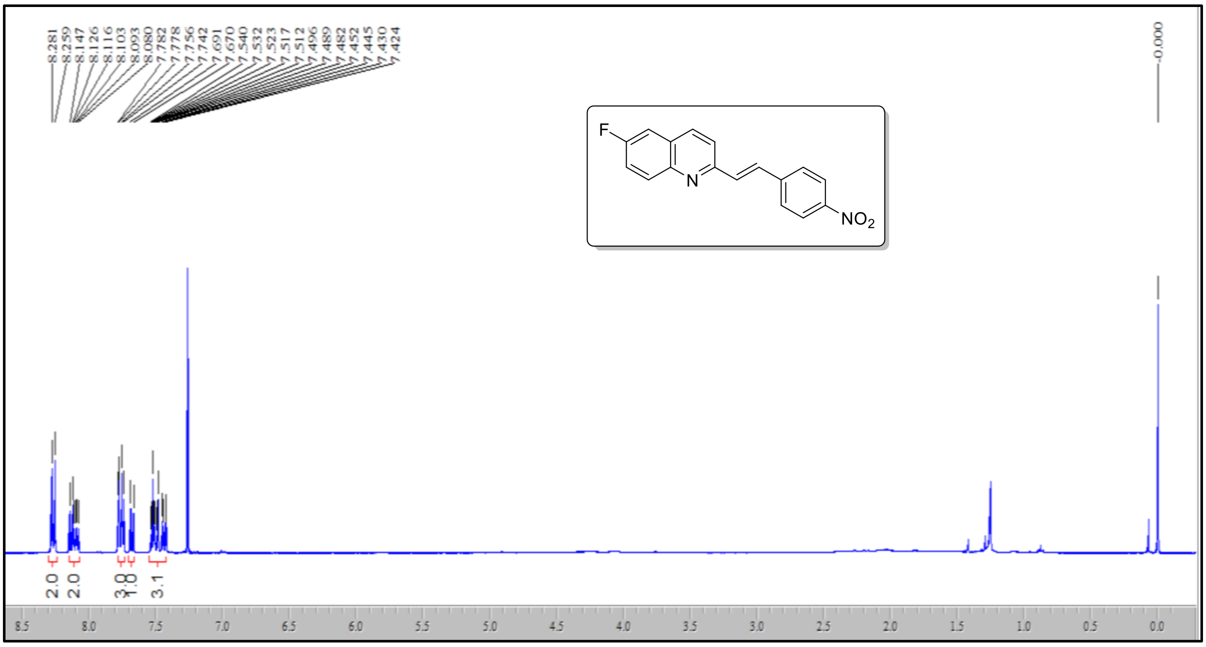

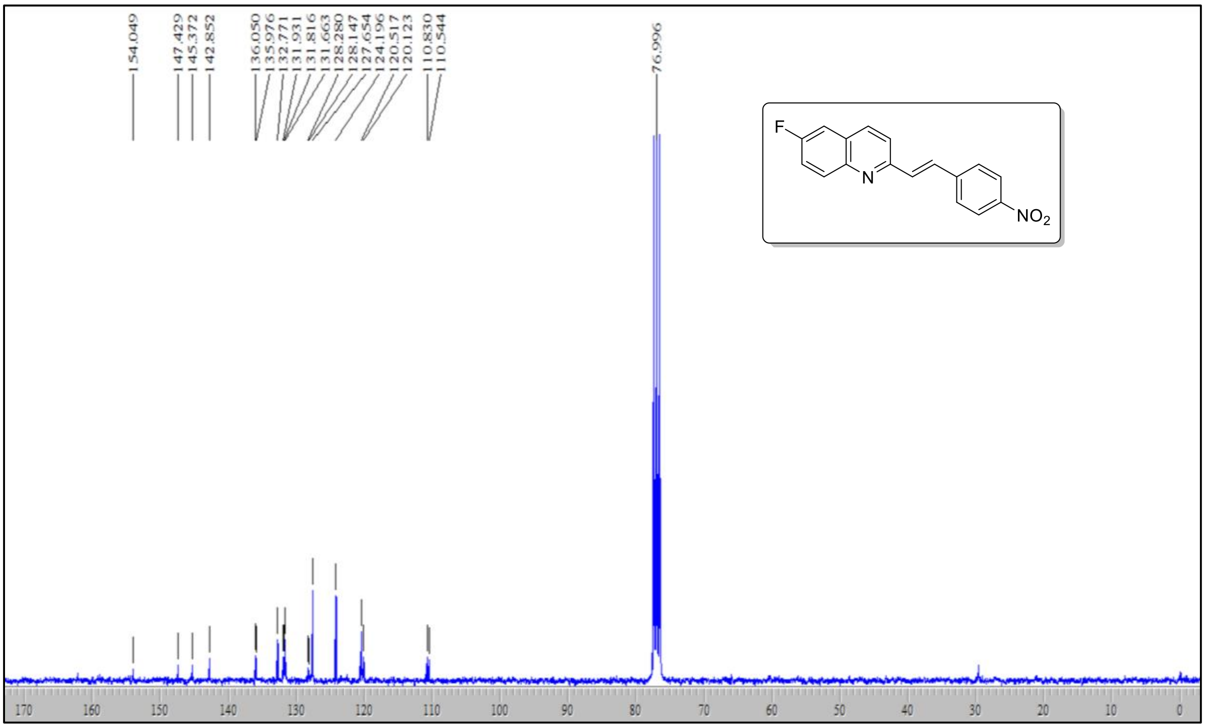

4. References

1. Ortalm, A.; Rathousky, J.; Ekloff, G. S.; Zukal, A. *Microporous Mater.*, **1996,** *6,* 171-180.
2. Dan, M.; Gang, H.; Shengying, W.; Xin, L.; Jianjun, Y.; Limin, W. *Eur. J. Org. Chem.,* **2014**, *2014,* 3009–3019.
3. Wang, F. F.; Luo, C. P.; Wang, Y.; Deng, G.; Yang, L. *Org. Biomol. Chem.*, **2012**, *10,* 8605–8608.
